# Micro-/nano-voids guided two-stage film cracking on bioinspired assemblies for high-performance electronics

**DOI:** 10.1038/s41467-019-11803-8

**Published:** 2019-08-27

**Authors:** Weining Miao, Yuxing Yao, Zhiwei Zhang, Chunping Ma, Shengzhe Li, Jiayue Tang, He Liu, Zemin Liu, Dianyu Wang, Michael A. Camburn, Jen-Chun Fang, Ruiran Hao, Xinyu Fang, Shuang Zheng, Nan Hu, Xiaoguang Wang

**Affiliations:** 1 Technical Institute of Physics and Chemistry, Chinese Academy of Sciences, Beijing, China; 2 University of Chinese Academy of Sciences, Beijing, China; 3 Department of Chemistry and Chemical Biology, Harvard University, Cambridge, MA USA; 4 Department of Civil, Environmental and Geodetic Engineering, The Ohio State University, Columbus, OH USA; 5 Department of Mechanical Engineering, Imperial College London, London, SW7 2AZ UK; 6 Beijing National Laboratory for Molecular Sciences, Institute of Chemistry, Chinese Academy of Sciences, Beijing, China; 7 College of Chemistry, Jilin University, Changchun, China; 8 William G. Lowrie Department of Chemical and Biomolecular Engineering, The Ohio State University, Columbus, OH USA

**Keywords:** Surfaces, interfaces and thin films, Materials for devices, Mechanical properties

## Abstract

Current metal film-based electronics, while sensitive to external stretching, typically fail via uncontrolled cracking under a relatively small strain (~30%), which restricts their practical applications. To address this, here we report a design approach inspired by the stereocilia bundles of a cochlea that uses a hierarchical assembly of interfacial nanowires to retard penetrating cracking. This structured surface outperforms its flat counterparts in stretchability (130% versus 30% tolerable strain) and maintains high sensitivity (minimum detection of 0.005% strain) in response to external stimuli such as sounds and mechanical forces. The enlarged stretchability is attributed to the two-stage cracking process induced by the synergy of micro-voids and nano-voids. In-situ observation confirms that at low strains micro-voids between nanowire clusters guide the process of crack growth, whereas at large strains new cracks are randomly initiated from nano-voids among individual nanowires.

## Abstract (web summary)

Metal film-based electronics usually fail via uncontrolled cracking at small strains. Here, the authors design a micro-/nano-structured surface with bundled flexible nanowires onto which a metal film can stretch more than a regular flat surface, retard penetrating cracks and remain sensitive.

## Introduction

Heterogeneities on the micro-/nanoscale in solids, such as voids and strain-induced cracks, can significantly influence bulk electronic, optical, and mechanical properties, which offers the basis of a new class of flexible devices, ranging from wearable electronics^1–6^ to mechanochromisms^7–9^ to microscale patterning^10–13^. Specifically, metal film-based wearable electronic devices, while promising, typically involve use of the nature of metal ductility, making them a fast and highly sensitive platform for locomotion monitoring. Mechanical strain-induced penetrating cracking, which refers to formation of cracks propagating perpendicularly to the strain and running throughout the whole conducting metal film, however, causes low tolerable strains (maximum detectable strain at which penetrating cracks form and disable the sensor) of these electronics and thus inherently limits their application for monitoring full range of activities. For example, numerous studies have shown that vigorous movements (e.g., >30% uniaxial strain) can disable the Pt film-based wearable electronics^1,14^. Although past studies have investigated the process of crack initiation and penetration^6,10,15–17^, manipulation of crack retardation to impart the metal film-based wearable electronics with ultrastretchability as well as high sensitivity has not yet been described.

To address the aforementioned issue, we seek solutions in nature with examples of structural materials and systems with hierarchical design. One of the examples is a cochlea system of an inner ear that uses acoustic-responsive hierarchical assemblies of stereocilia at surfaces of tympanic membranes to efficiently convert acoustic signals into electrochemical activities and reach a compromise between detection range and sensitivity^18–22^ (Supplementary Fig. 1 and Supplementary Note 1). Other hierarchical designs have also inspired recent advances in soft electronics^23–29^ and materials^30–34^ with programmable shape transformation and stretchability at a wide range of scales.

Inspired by the hierarchical design methodology both in nature and engineered systems, here we report a design that uses hierarchical assembly of interfacial nanowires to retard penetrating cracking and significantly increase the stretchability of metal film-based sensors. Finite element simulations, characterization of the morphologies of both structured and flat surfaces under different strains coated by nanometer-thick metal films, and conductivity measurements, reveal that this improvement is attributed to a two-stage crack-generation synergy. At low strain, crack generation occurs along micrometer-sized voids between the nanowire clusters, and at high strain, subsequent crack initiation originates from nanometer-sized voids among the individual nanowires within the clusters. This process is similar to the central role of stereocilia bundles in the cochlea system. This two-stage cracking process allows high stretchability (130% strain) and high sensitivity (Gauge Factor of 107.45, minimum detection of 0.005% strain) to be combined into a single sensor. Further, we illustrate the utility of this bioinspired approach by fabricating wearable electronics such as sound detectors and soft robotics actuation monitors, which achieve high stretchability and retain high sensitivity compared to conventional metal film-based electronics.

## Results

### Fabrication of the hierarchical nanowire assemblies

**Fig. 1:**
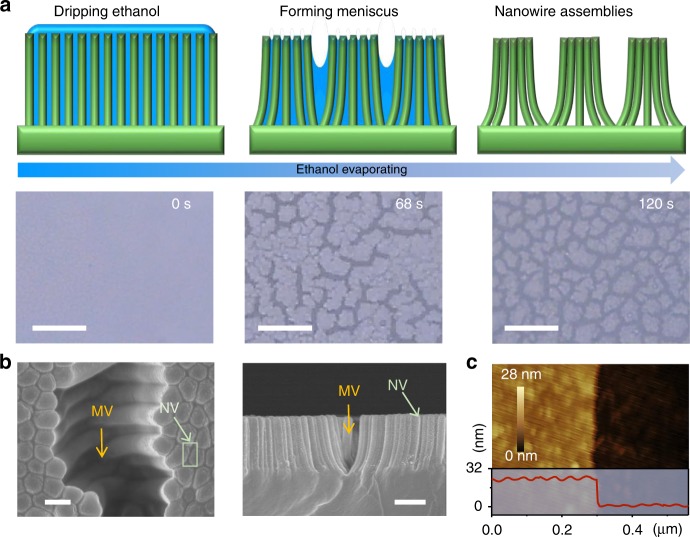
Bioinspired hierarchical assembly of nanowires. **a** Schematic illustrations and corresponding optical micrographs of capillary force-induced assembly of nanowires (scale bar, 20 μm). **b** SEM images of micro-voids between nanowire clusters and nano-voids among individual nanowires within a single nanowire cluster (scale bar in left and right figures are 500 nm and 2 μm, respectively). MV (yellow arrow) and NV (green arrow) represent micro-voids and nano-voids, respectively. **c** Atomic force microscopy (AFM) topography image of the deposited 24-nm-thick Pt film

In our experiments, we fabricated structured surfaces consisting of nanowire assemblies that mimic the stereocilia bundles in the cochlea. First, we prepared a polydimethylsiloxane (PDMS) nanowire array by using soft lithography templated by a porous anodic aluminum oxide (AAO) mold with a pore diameter of 400 nm and a center-to-center spacing of 450 nm (see Supplementary Figs. 2 and 3). PDMS was selected as the material for flexible polymeric substrates due to its excellent elasticity, biocompatibility and chemical stability^35–38^, and ethoxylated polyethylenimine (PEIE) was doped in PDMS to enhance the stretchability^39^ (Supplementary Figs. 4 and 5). Next, the formed PDMS nanowire array with a nanowire diameter of 400 nm was immersed in ethanol. As ethanol evaporated, the nanowires bent and clustered by lateral capillary force, resulting in the formation of bundles of close-packed nanowires with nearest neighbor center-to-center spacing between nanowire tips ~400 nm (see Fig. 1a, Supplementary Note 2, Supplementary Figs. 6–9, and Supplementary Movie 1 for details). As shown in the scanning electron microscopy (SEM) images of the surface morphology (Fig. 1b), native micrometer-sized voids between nanowire clusters and nanometer-sized voids among individual nanowires are randomly distributed over the entire nanostructured substrate. Finally, a 24-nm-thick Pt film was coated on this hierarchically structured surface (see Fig. 1c and Supplementary Fig. 10). We comment here that the Pt element was only deposited on the surface of the nanowire assemblies and would not penetrate into the nano-voids between individual nanowires within the nanowire clusters (see Supplementary Fig. 11).

### Geometric effect of stretched bioinspired nanowire assembly

**Fig. 2:**
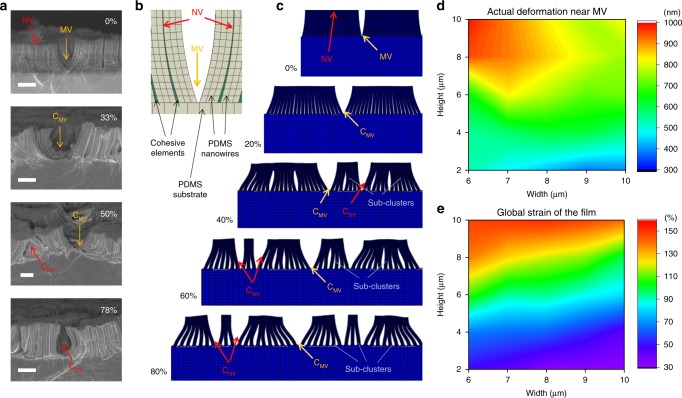
Simulation of cracking of nanowire-structured surfaces guided by MVs and NVs. **a** SEM images of cracking induced by MVs (yellow arrow) and NVs (red arrow) of Pt films (scale bar, 2 μm). C_MV_ (yellow arrow) and C_NV_ (red arrow) represent cracks initiated from MVs and NVs. **b** Key features of nanowire assembly in FE modeling. **c** Evolution of hierarchical assembly of 4-μm-length nanowires as a function of strain (See Supplementary Movie 2). **d**, **e** Influence of nanowire length and cluster size on (**d**) MV-induced cracking or (**e**) global strain of the nanowire-structured surfaces

The premise of our design is that the presence of hierarchical assembly of nanowires has the potential to retard penetrating cracking under large mechanical strains. We used SEM to image the morphology of nanowires at different stages of stretching process. Inspection of Fig. 2a reveals that the hierarchical assembly of 4-μm-length nanowires exhibited a two-stage sequential cracking process starting from stretching at micro-voids (MVs) followed by de-bonding of nanowires at nano-voids (NVs). Eventually, a micro-sized crack developed within a single nanowire cluster and led to two sub-clusters at a high strain. We note here that we use the term ‘voids’ and ‘cracks’ to describe the gaps between nanowires before and after applying strain, respectively (Supplementary Figs. 12 and 13).

To understand this observed phenomenon, we performed nonlinear simulations through the commercial finite-element (FE) software ABAQUS. We selected a 2D FE model that is well approximated to plane stress conditions (i.e., the film is loaded uniformly through the thickness considering the film width is much larger than its thickness) and computationally efficient enough to simulate the stretching process of the film. According to the experimental observation, all our FE models were designed to consist of two nanowire clusters with nanowire lengths of 4 μm and cluster sizes of 8 μm (20 nanowires per cluster, and 450 nm center-to-center spacing between the base of the nanowires) on a 6-μm-thick rectangular PDMS substrate (see Fig. 2b, Supplementary Note 3 and Supplementary Fig. 14 for details). We used cohesive elements in the FE model to assume the adhesive force between nanowires. Nanometer-sized Pt layers were excluded in our model for simplicity. The PDMS substrate was subjected to horizontal extension at the both ends, and we terminated the model when six sub-clusters formed at the surface. We notice here that our model captures the characteristic parabolic shape of the nanowires at the edge of the nanowire bundles.

Figure 2c and Supplementary Movie 2 show the simulation results of the stretching process in our FE model. For strains up to 20%, the applied strain was mainly accommodated by increasing the gaps between neighboring clusters (i.e., MVs), which is consistent with the signatures that we observed in the morphology of nanowires at low strains. We name this process the MV-induced cracking stage (C_MV_). When the strain reached around 40%, the nanowires within the same bundle began to de-bond. As the applied strain increased, the cracking initiated across the entire nanowire-structured surface and propagated toward NVs, leading to the creation of NV-induced cracks (C_NV_) and several sub-clusters consisting of a smaller number of nanowires. To this end (~80% strain), two clusters cracked into six sub-clusters, a signature consistent with the behavior of NV-induced cracking. Theoretically, the enhancement of the stretchability of our film due to the presence of nanowires can be explained by a study in the field of condensed matter physics that breaking random bonds within the material will lead to a diffusive failure other than major cracks^40^.

To provide insight into the role of the nanowire cluster, we further performed a parametric study to investigate the effect of the nanowire length and the cluster size on the local strain of the MVs and global strain of the whole film, respectively (see Supplementary Table 1 for details). We measured the extension at 600 nm above the MV. As shown in Fig. 2d, as either the nanowire length increased or the cluster size decreased, the size of the cracks induced by the MV increased. Moreover, Fig. 2e shows that the nanowire length has a more pronounced impact on the stretchability of the film compared to the size of the cluster. Overall, our numerical results demonstrate the characteristic two-stage cracking behavior guided by both MVs and NVs at the nanowire-structured surface, which is in good qualitative agreement with our experimental observations. We note here that the accuracy of our FE modeling can be improved by considering the stochastic nature among cohesive elements with the randomness of material properties.

### Retarding penetrating cracks by the synergy of MVs and NVs

**Fig. 3:**
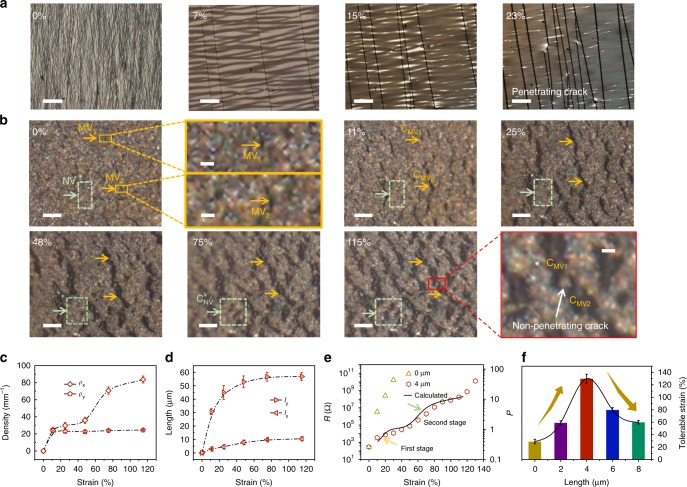
Influence of strains on morphology and electrical resistance of Pt films. **a**, **b** In-situ imaging of cracking process of Pt films coated on (**a**) a flat surface or (**b**) a nanowire-structured surface at different strains, respectively (two MVs and corresponding C_MV_ are marked with yellow arrows or box; NV region and corresponding C_NV_ are marked with greed dashed box; scale bar in **a**, 200 μm; scale bar in **b**, 20 μm; scale bar of enlarged figures of 0% and 115% strain in **b**, 5 μm). **c**, **d** Linear density and length of cracks of the Pt film coated on hierarchical assemblies of 4-μm-length nanowires plotted as a function of strains. Subscripts x and y represent the directions parallel and perpendicular to the stretch, respectively. Error bars, s.d. (*n* = 20). **e** Electrical resistance of the Pt film and calculated *P* (*ρ*_x_*l*_x_ / (*l*_0_-*ρ*_y_*l*_y_)) as a function of strains (see Supplementary Note 4 for details). **f** Tolerable strain as a function of nanowire length. Error bars, s.d. (*n* = 5)

To further test the hypothesis predicted by the FE model, we performed additional measurements to provide experimental evidence regarding the mechanism of two-stage cracking guided by the MVs and the NVs and further insights into the correlation between the strain and the electrical resistance of the Pt film-coated nanowire-structured surfaces. We imaged the morphological evolution of Pt film over a wide range of strains (0–140%). As shown in Fig. 3a, the cracks were first initiated at random sites on the Pt film at a flat surface under small strain (~7%). As the applied strain further increased, the formed cracks propagated rapidly in the direction vertical to the stretch and eventually penetrated throughout the whole Pt film, as indicated by a significantly high electrical resistance, which is consistent with previous studies^10^.

Next, we applied strain to the surface with assemblies of 4-μm-length nanowires (Fig. 3b). In-situ optical microscopy imaging was performed to determine whether the cracks were induced by MVs or NVs. As the uniaxial tensile strain to the substrate was applied, cracks started to initiate at the location of MVs where the stress was larger than other regions (i.e., NVs between the individual nanowires) due to a weak nanowire-retarding effect. For example, as shown in Fig. 3b, prior to 11% strain, cracks C_MV1_ and C_MV2_ were initiated from MV_1_ and MV_2_, respectively. Furthermore, the sizes of MV-initiated crack lengths, along the directions either parallel or perpendicular to the stretch, increase with the strain up to ~48%, whereas the NVs remain intact within the same range of strain, as evidenced in Fig. 3b. Interestingly, when the strain reached a critical threshold (~48%; Fig. 3b), a second-stage crack initiation stemmed from rather the NVs between individual nanowires within nanowire clusters than the micrometer-sized counterparts between the nanowire clusters. We comment here that we still observed bridging of neighboring cracks instead of penetrating cracking even at 115% strain (Fig. 3b), which has not been achieved in conventional flat metal films^7^.

To fully understand the underlying cause of crack retardation by nanowire assemblies, we characterized the numerical density of cracks as a function of strain (Fig. 3c). *ρ*_x_, the linear density of cracks along the strain direction, increased at a high rate with strain in the range of 0–10% and 50–80%, corresponding to the crack initiation from MVs and NVs, respectively. On the other hand, *ρ*_y_, the linear density of cracks perpendicular to the strain, increased until plateauing when the strain reached ~10%, corresponding to crack merging in the direction vertical to the strain. Moreover, the average lengths of the cracks parallel and perpendicular to the strain (denoted by *l*_x_ and *l*_y_, respectively) increased steeply up to a threshold value (~50%) above which the rate of change of *l*_x_ and *l*_y_ greatly diminished (or even became negative) due to the second stage of crack initiation by NVs, as shown in Fig. 3d. These results lead us to conclude that in contrast to the rapid penetrating cracking on the flat surface, the hierarchical assembly of nanowires retarded penetrating cracking in a two-step mode – crack initiation from MVs and NVs at the onset strains of 0% and ~50%, respectively, which is in qualitative agreement with our FE modeling.

As described in the Introduction, we sought to manipulate the crack formation to design a metal film-based electronic device with both ultrastretchability and high sensitivity. Therefore, we next determined if the electrical properties of the Pt film coated on the nanowire assemblies correlate strongly with the structure of the cracks. The results of the electrical conductivity measurements are shown in Fig. 3e (see Supplementary Figs. 15 and 16 for details). The measured resistance was shown to increase at a higher rate with the strain in the range of 0–10% and 50–80%, which coincides with in-situ observation of the two-step crack initiation from MVs and NVs, respectively. We notice here that the electrical resistance of the metal film coated on the nanowires correlates strongly with *ρ*_x_, which is consistent with past studies of conventional flat metal films^1^. To provide additional support for our experimental observations, we derived a simple model to describe the relationship between crack generation and the electrical properties of the Pt film. As shown in Fig. 3e, the calculated result captures the essential change in the strain-dependent resistance (i.e., two-stage mode), which is in good qualitative agreement with our experiments (see Supplementary Note 4 and Supplementary Fig. 17 for details of both parameter *P* and the model).

Next, we investigated the effect of nanowire length on the tolerable strain of the nanowire-structured Pt films (Supplementary Fig. 18). Inspection of Fig. 3f reveals that the tolerable strain of the nanowire-structured Pt films increased with the length of the nanowire, and the maximum value reached 130% (for 4-μm-length nanowire), which is at least three times larger than that for the non-structured Pt film (~30% strain). Surprisingly, further increases in nanowire length caused a decrease in the tolerable strain of the nanowire-structured Pt films, which contradicts the estimations from our FE models. As shown in Fig. 3f, the tolerable strains of 6 and 8-μm-length nanowire-structured Pt films were 80% and 60%, respectively. We attribute this observed trend to the synergy of MVs and nanowire interactions. On one hand, longer nanowires result in larger bundles with fewer MVs (Supplementary Fig. 19), which would induce larger MV-induced cracks during the first stage (i.e., MV-induced cracking). On the other hand, strong adhesive forces between long nanowires delay the onset strain of the second stage (i.e., NV-induced cracking), which suggests that MV-induced cracks would be significantly stretched and easily connected to form penetrating cracks (see Supplementary Fig. 20 for details). Overall, these results demonstrate that the hierarchical micro-/nanostructures present opportunities to achieve high levels of stretchability in metal film-based electronics.

### Ultrastretchable and highly sensitive electronics

**Fig. 4:**
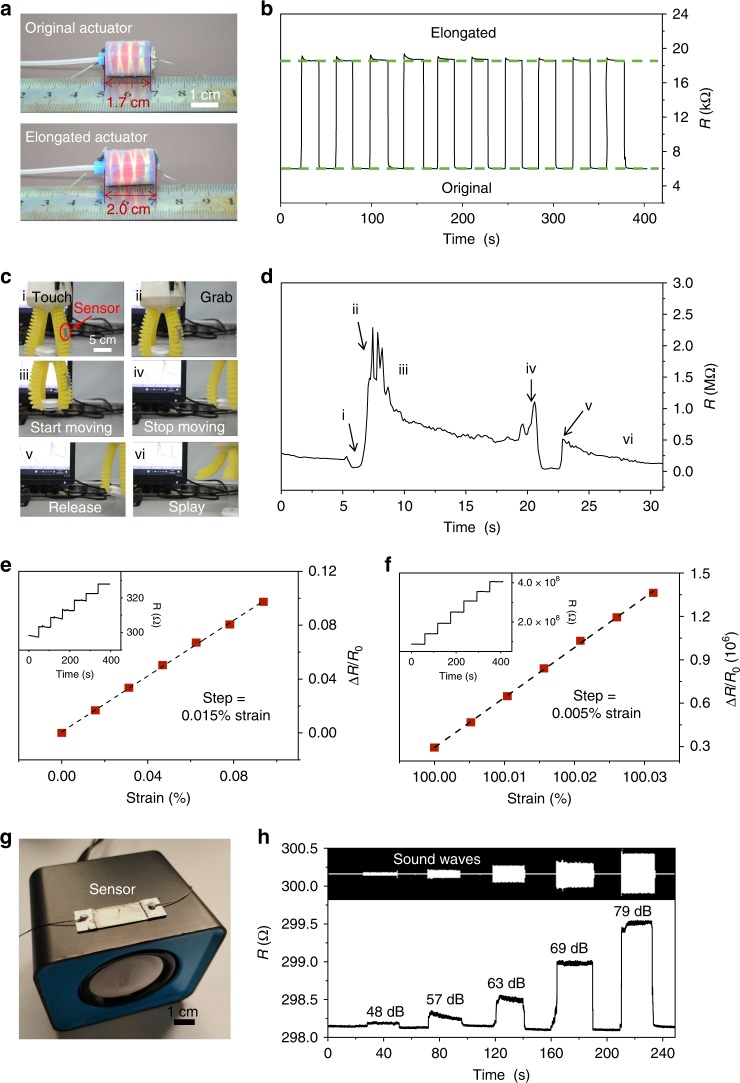
Ultrastretchable and highly sensitive electronics. **a** A 1.5-cm-length 1D actuators installed with our metal film-based electronics. **b** The electrical resistance of the metal film sensor as a function of actuator deformation. **c** Nanowire-structured Pt films for detection of the soft gripper’s locomotion. **d** Variation of the resistance of the metal film sensor in the process of grabbing-holding-releasing objects by the soft gripper (see Supplementary Movie 3 and 4). **e**, **f** Δ*R*/*R*_0_ of cracked Pt film on nanowire clusters versus strain within the range of (**e**) 0–0.1% or (**f**) 100–100.03%. Insets show the corresponding resistance as a function of strain. **g** A nanowire-structured Pt film-based sensor mounted on a loudspeaker. **h** Influence of sound intensity on the resistance of the metal film sensor (See Supplementary Movie 5). The length of the nanowire is 4 µm

We end this paper by exploring the above, bioinspired strategy as a general and simple method for the fabrication of highly sensitive and ultrastretchable soft electronics with potential applications in sound detection and soft robotics actuation monitors. First, a metal film-based electronic device consisting of an assembly of 4 µm-length nanowires was fixed onto a 1.5 cm-length 1D actuator (Fig. 4a and Supplementary Fig. 21). As evidenced by the detection of numerous cycles of deformation of the 1D actuator in Fig. 4b and Supplementary Fig. 22, our electronic sensor exhibited good reversibility with little deterioration compared to previous elastomer-based metal film sensors (see details in Supplementary Note 5 and Supplementary Fig. 22, 23)^14,41,42^. Secondly, the electronic device was attached to a soft gripper with a 10-cm-length claw to monitor a wide range of its activities (Fig. 4c and Supplementary Fig. 24). The resistance shows obvious increases and decreases when the claw splayed outwards and gripped inwards, respectively (Supplementary Movie 3 and Supplementary Fig. 25). In addition, as shown in Fig. 4c, d and Supplementary Movie 4, all locomotive modes of the soft gripper during consecutive interactions with an object including touching, grabbing, starting motion, stopping motion, releasing, and eventually splaying, were clearly distinguished by the device. More importantly, our nanowire-structured Pt film was measured to be sensitive to tiny deformations over a wide range of strain. As shown in Fig. 4e, f, the surface was able to detect a minimum strain of 0.015% and 0.005% at both small (0%) and large (100%) strains, corresponding to a gauge factor (defined as Δ*R*/(*εR*_0_), where *ε* represents the applied strain) of 107.5 to 2.6 × 10^5^, respectively (Supplementary Fig. 26). These results lead us to conclude that the nanowire-structured Pt film sensors maintain a high sensitivity over a wide range of strain and outperform most of the currently reported metal and other conductive material-based sensors (see Supplementary Table 2).

In past studies, unique substrate geometries and shapes, such as serpentine^43–45^, fracture^46^, and mesh^47,48^, have been designed to enlarge the detection range of metal film-based sensors. We further investigated the effect of the nanowire structure and the substrate geometry by fabricating flat, nanowire-structured, serpentine-shaped Pt film sensors (see Supplementary Note 6 and Supplementary Figs. 27–29). While the serpentine-shaped design leads to an increase in the stretchability of the Pt film sensors, the serpentine structure overall lowers the minimum strain detection and gauge factor of the devices, as evidenced in Supplementary Table 3. We also demonstrated that the device can be highly sensitive to other types of external stimuli. As evidenced in Fig. 4g, h and Supplementary Movie 5, we attached the electronic device onto a loudspeaker, and it exhibited high sensitivity (minimum sound intensity of 48 dB) and good reversibility (see Supplementary Fig. 30 and Supplementary Movie 6) to acoustic signals. Overall, these results hint that these hierarchically nanowire-structured surfaces may find uses in technologies that require high levels of both stretchability and sensitivity^49–53^.

## Discussion

To conclude, we have achieved crack retardation in a metal film by structuring surfaces with a hierarchical assembly of nanowires based on principles derived from nature, e.g., the deflection of stereocilia of cochlea in response to sounds. Specifically, two-stage crack initiation from MVs between the nanowire clusters at low strains and subsequently from NVs among individual nanowires at large strains result in ultrastretchability of the surface. Furthermore, the electrical resistance of the metal film correlates strongly with a linear density of cracks along stretching and provides a sensitive measure of small deformations over a broad range of strain (0–130%). Overall, the combined properties of the nanowire-structured metal film reported in this paper (i.e., ultrastretchability and high sensitivity) are difficult to achieve in conventional flat counterparts, and in the long term, such structured surfaces have the potential to be useful in applications such as wearable electronic devices for motion detection and health monitoring. The hierarchical micro-/nanostructures have the potential to provide fundamental insights into the roles of heterogeneities in the properties of metallic material systems. Moreover, the methodology reported in this paper can likely be extended to more complex structures and materials, leading to more exotic mechanical and optical properties. Future efforts will seek to develop an advanced 3D FE model to quantify the relationship between the applied strain and the morphology of the nanowire assembly.

## Methods

### Fabrication of Pt film-coated nanowire clusters

First, we mixed 30 μL of PEIE solution (80% v/v; molecular weight is 70,000 g/mol; Sigma-Aldrich) with PDMS precursor mixture, which consisted of 10 g of Sylgard 184 precursor and 1 g of curing agent (Dow corning). The mixture was stirred for 10 min and then subjected to vacuum for 1 h to remove small air bubbles. Second, 0.16 mL of the mixture was applied to a 20 mm × 20 mm AAO (Hefei Pu-Yuan Nano Technology Ltd.) nanopore template, which consists of an array of 400-nm-in-diameter pores with 450 nm center-to-center spacing and 2–8-μm-height. Third, the mixture was settled for 30 min, followed by thermal curing at 90 °C for 3 h. Fourth, the as-prepared PDMS was soaked in 1 M hydrogen chloride solution at 80 °C to remove the AAO mold, and the obtained PDMS nanowires were rinsed by water. Fifth, 5 mL of ethanol was added to the nanowires, and then the surface was settled under ambient environment for 10 min to completely evaporate the ethanol, resulting in an assembly of nanowires. Finally, 24 nm-thick Pt was deposited onto the clustered nanowires within a LEICA EM SCD 500 vacuum sputtering plating machine at 25 mA for 1900 s.

### Surface morphology characterization

The evolution of surface morphology during the evaporation of ethanol was obtained by using an optical microscope (Vision Engineering Co.) equipped with a charge-coupled device (CCD) camera connected to a computer. The transmission of the nanowire clusters was measured by collecting UV-vis absorption spectra with a Shimadzu UV-3600 spectrometer at room temperature. The morphologies of the cracks at the nanowire-structured surfaces were investigated via Hitachi S-4800 SEM at an acceleration voltage of 5 kV. EDS was scanned linearly via Hitachi S-4800 SEM at an acceleration voltage of 10 kV. X-ray photoelectron spectroscopy (XPS) elemental mapping was measured on a Thermo Scientific ESCALab 250Xi using 200 W monochromatic Al Kα radiation. The spot size of X-ray is 500 μm and the hydrocarbon C1s line at 284.8 eV from adventitious carbon is used for energy referencing. AFM imaging was performed on a Bruker Dimension Icon AFM instrument, and a chrome mask was used to gain the margin of the Pt film coated on a flat surface.

### Electrical conductivity measurement

The current-voltage characteristics of the metal film were obtained by a Keithley 6487 picoammeter. The electronic device was stretched by attaching the electronic to a computer-controlled motion controller (Taida Machinery Equipment Co., TH16–57). The acoustic signals that used in our experiments were 600 Hz sinusoidal waves with different amplitudes corresponding to sound intensities ranging from 48 dB to 79 dB, which were generated by a loudspeaker controlled by a frequency sound generator. The sound intensity was determined by using a Smart Sensor type AR844 digital sound level meter at the position of 10 cm in front of a loudspeaker. Silicone adhesive was used to attach the metal film device to either a 1D actuator or a soft robotic claw. Details regarding fabrication of the 1D actuator and the soft robotics can be found in Supplementary Information.

## Supplementary information

Supplementary information

Description of Additional Supplementary Files

Supplementary Movie 1

Supplementary Movie 2

Supplementary Movie 3

Supplementary Movie 4

Supplementary Movie 5

Supplementary Movie 6

## Data Availability

The authors declare that the main data supporting the findings of this study are contained within the paper. All other relevant data are available from the corresponding author upon reasonable request.
