# Supplementary material for: Micro-/nano-voids guided two-stage film cracking on bioinspired assemblies for high-performance electronics
Source: Nat Commun. 2019 Aug 27;10:3862. doi: 10.1038/s41467-019-11803-8 (PMC6711965; doi:10.1038/s41467-019-11803-8)
Supplement: Supplementary file 1 — Supplementary information [file 41467_2019_11803_MOESM1_ESM.pdf]

Supplementary Information

Micro-/nano-voids guided two-stage film cracking on bioinspired assemblies for high-performance electronics

Miao et al.

Supplementary Note 1. Process of acoustic signal detection by a cochlea.

Stereocilia bundles distributed on hair cells are key for converting mechanical stimuli to electrical signals. As sound waves travel through a cochlea, they induce the reversible vibration of stereocilia clusters and the subsequent nano-deflection of individual stereocilia (Supplementary Fig. 1), which enlarges the nano-voids, stretches the filamentous structures connecting the tips of neighboring stereocilia, and gives rise to the activation of ion channels for ultrasensitive signal transduction[1, 2]. When exposed to a high intensity acoustic signal (<130 dB), however, the mobility of individual stereocilia would be restrained by the bundle structure, thus preventing the degeneration of the hair cells and subsequent tinnitus or hearing loss[3, 4]. Accordingly, the hierarchical micro-/nanostructures of stereocilia assemblies and the associated cooperative movements between individual stereocilia and their clusters enable the cochlea system to tolerate high acoustic intensity (<130 dB) as well as to be ultrasensitive[5].

Supplementary Note 2. Formation of bioinspired hierarchical assembly of nanowires.

Fabrication of hierarchical assembly of nanowires is shown in Supplementary Fig. 6. Nanowires were first dispersed uniformly in ethanol. As ethanol evaporated, micrometer-sized meniscuses appeared when the top surface of ethanol receded to the top of the flexible nanowires, resulting in clustering of nanowires and thus formation of micro-voids between the nanowire clusters. Further evaporation induced the climbing of the trapped ethanol within the clusters due to the axial force from curvature pressure differences between meniscuses at the top and bottom sides. Finally, with ethanol completely evaporated, nanowires were tightly bound, giving rise to nano-voids between individual nanowires within the cluster.

Supplementary Fig. 7 details the force underlying the process of nanowire assembly during ethanol evaporation. Lateral force (F_C) arises due to formation of small meniscuses connecting neighboring nanowires[6-9] and can be estimated by Supplementary Equation 1:

$$F_C = 2\pi\gamma R^2\cos^2\theta\left(\sqrt{\frac{2}{(d_0-2\delta)^2-4R^2}}+\sqrt{\frac{1}{2(d_0-2\delta)^2-4R^2}}\right) \quad (1)$$

where γ, R, d_0 and δ represent the surface tension, radius of nanowires, center-to-center distance between neighboring nanowires and displacement of nanowire tips, respectively. In addition, θ is the intrinsic contact angle of ethanol on a flat PDMS substrate (see Supplementary Fig. 8).

F_C is resisted by the elastic restoring force (F_E)[6, 10], which can be expressed as:

$$F_E = \frac{3\sqrt{2}\pi E R^4\delta}{4h^3} \quad (2)$$

in which h is the length of nanowires, and E is the Young's modulus that was measured to be 0.24 MPa (see Supplementary Fig. 4). For the nanowires to bend and assemble (i.e., $\delta = (d_0 - 2R)/2$), F_C must overcome F_E. We note here that nanowires with any lengths (i.e., 1-4 μm) can assemble into clusters due to the low Young's modulus of PDMS. Thus, as nanowire clustering proceeds, the spacing gradient arises along the length of the nanowires, resulting in the climbing of the trapped ethanol. The difference in Laplace pressure along the nanowire length can be calculated as:

$$\Delta P = \frac{4\gamma\cos\theta_1}{d_1} - \frac{4\gamma\cos\theta_2}{d_2} \quad (3)$$

where θ_1, θ_2, d_1 and d_2 are the advancing contact angle, receding contact angle, and diameters of the top and bottom menisci, respectively. Upon full evaporation of ethanol, the assembly of nanowires causes a decrease in transparency[11], as evidenced in Supplementary Fig. 9.

Supplementary Note 3. Details on finite element simulations.

All our FE analysis was performed through the commercial software ABAQUS. A 2D shell model was built to simulate the deformation of a PDMS nanowire cluster connected by cohesive elements. The PDMS nanowire cluster and substrate were constructed using 4-node bilinear plane stress quadrilateral element (CPS4), while the cohesive region between nanowires were built with 4-node two-dimensional cohesive element (COH2D4). Based on the mechanical test of our PDMS film and other recent studies[12-14], we used the Mooney-Rivlin model to define PDMS with hyperelastic properties (C10=0.201, C01=0.041, D1=0). Cohesive elements are widely used to model adhesive force or debonding effect in ABAQUS[15-17]. Six different cohesive material properties were selected at various locations among the cluster because both trial simulation and experimental observations have indicated an uneven distribution of adhesive forces between nanowires. The specific properties and distribution of cohesive elements is given in Supplementary Fig. 14. The ABAQUS/Standard general static solver was used considering geometric nonlinearity (NIgeom option on). The dissipated energy fraction was selected with the automatic stabilization option and a specified coefficient of 00002. General contact was used with frictionless and normal "hard" contact properties. Only vertical supports were applied at the bottom of the PDMS substrate. Our simplified FE model is acceptable for the purpose of capturing the key signatures of the two-stage cracking process. We note here that our FE model only presents a possible distribution with two weaker regions (C11-C12 and C21-C22) among one cluster, while real adhesive forces might have a gradient along the nanowires and across the clusters during the ethanol evaporation process.

Supplementary Note 4. Details on calculation of resistance of Pt film.

Here we develop a model to relate the resistance of stretched Pt film to the statistics of cracks. In this model, penetrative cracks are considered to exhibit infinite resistance. As detailed in Supplementary Fig. 17, R^N and R_{ij} represent the resistance of Pt film between two cracks perpendicular and parallel to the stretch, respectively. The original resistance R_0 can be expressed as:

$$R_0 = R^1 + R^2 + \cdots + R^N \quad (4)$$

Upon the stretching of the Pt film, strain-induced cracks cause an increase in the resistance, which can be written as:

$$R = R^1 + R^2 + \cdots + R^N + \sum_{j=1}^{N} \left(\sum_{i=1}^{M} \frac{1}{R_{ij}}\right)^{-1} \quad (5)$$

where R_{ij} can be further described by:

$$R_{ij} = \frac{r l_\mathrm{x}}{h_\mathrm{Pt} l'} \quad (6)$$

in which the width of the cracks is denoted as l_x, r is the electrical resistivity of Pt, and the thickness (h_Pt) of the Pt film is assumed to be a constant. In addition, l' represents the average distance between adjacent cracks from different rows:

$$l' = \frac{l_0 - \rho_\mathrm{y} l_\mathrm{y}}{\rho_\mathrm{y} - 1} \quad (7)$$

in which l_0 is the unit length we defined (in this case, 1.5 mm), l_y is the average length of cracks, and ρ_y is the linear density of cracks perpendicular to the strain.

In a unit area (1.5 mm × 1.5 mm), the resistance change can be estimated as:

$$\Delta R = R - R_0 \approx \frac{\rho_\mathrm{x}}{\rho_\mathrm{y}} \cdot R_{ij} \quad (8)$$

where ρ_x is the linear density of cracks along the strain direction.

To better understand the relationship between the strain and the resistance, we introduce a new parameter P:

$$P = \frac{\rho_\mathrm{x} l_\mathrm{x}}{l_0 - \rho_\mathrm{y} l_\mathrm{y}} \quad (9)$$

Thus, we obtain:

$$\Delta R = \frac{\rho_\mathrm{x}}{\rho_\mathrm{y}} \cdot r \cdot \frac{l_\mathrm{x}}{h_\mathrm{Pt} \frac{l_0 - \rho_\mathrm{y} l_\mathrm{y}}{\rho_\mathrm{y} - 1}} \approx \frac{r \rho_\mathrm{x} l_\mathrm{x}}{h_\mathrm{Pt}(l_0 - \rho_\mathrm{y} l_\mathrm{y})} \propto P \quad (10)$$

Supplementary Equation 10 shows that the resistance change (ΔR) increases proportionately with P. We note that the resistance change per unit area is determined by the density, width and length of the cracks.

Supplementary Note 5. Cycleability of nanowire-structured metal film sensor.

In order to test the reversibility and durability of our device, we performed a long-term reversible loading test to our 4 μm-length nanowire-structured metal film sensor under 50% strain. As evidenced in Supplementary Fig. 22, our sensor exhibited good electrical stability and reversibility with no measurable fatigue over ~ 1,512 cycles, which suggests a significant improvement in the device performance compared with metal film sensors on flat surfaces[18-20]. We attribute such enhanced behavior to the interfacial nanowire assembly structure. Specifically, the nanowire clusters provide additional sites to release the strain in the metal film, resulting in accommodation of large deformations by the device. We note here that the measurable decay of the sensor appeared beyond ~ 1,512 cycles, which may result from the fatigue of the polymeric materials.

To provide insight into the origin of the outstanding reversibility and durability of our nanowire-structured sensors, we measured the hysteresis curve of the PDMS polymeric materials used to fabricate our metal film sensors. As shown in Supplementary Fig. 23, our PEIE-modified PDMS film exhibited relatively low hysteresis, giving rise to the excellent reversible characteristics of our sensor. We anticipate that the reversibility of the sensor can be further improved by using materials with ultralow hysteresis and self-healing properties.

Supplementary Note 6. Sensitivity and stretchability of nanowire-structured metal film sensors.

We compared the stretchability, gauge factor, and minimum strain detection of our nanowire-structured sensor with previous studies, as summarized in Supplementary Table 3. Metal films in a flat geometry exhibit an ultrahigh gauge factor of ~ 200 [21]. Past studies have reported the design of metal film sensors in a variety of geometries to reduce the stress in the stretchable substrate and thus significantly extend the stretchability of the device. However, such devices exhibited a low gauge factor (~1-10) with a minimum strain detection of ~ 2%, as summarized in Supplementary Table 3. In this work, we created hierarchical nanostructures at the stretchable surface to achieve both high stretchability and ultrasensitivity. Specifically, the gauge factor of our nanowire-structured metal film sensors reached 107.45 at the original length, which is the highest among all the listed metal film-based ultrastretchable sensors that can withstand more than 50% strain. In addition to the gauge factor, our sensor was able to detect a minimum strain of 0.015% and 0.005% at small (0%) and large (100%) strains, respectively.

To further provide insights into the role of nanowire assembly structure on the device performance, we fabricated serpentine-shaped sensors with/out surface nanowire structures by depositing a 24 nm-thick Pt film onto a flat serpentine substrate (called flat serpentine sensor; Supplementary Fig. 28a) and a serpentine substrate with assemblies of 4 μm-length nanowires (called nanostructured serpentine sensor; Supplementary Fig. 28c and 29). As shown in Supplementary Fig. 30, the maximum strains of flat and nanostructured serpentine sensors reached 80% and 240%, respectively. The enhanced stretchability of nanostructured serpentine sensors is attributed to the synergy of stress release by the macroscopic serpentine geometry and crack retardation effect associated with nanowire structures. These observations demonstrate that the macroscopic geometry and our surface structures can be combined harmoniously into one single sensor in order to obtain ultrastretchability.

Next, we measured the gauge factor of the flat serpentine and the nanowire-structured serpentine sensors to be 7.34 and 3.53, respectively, which are much smaller than that of our regular nanowire-structured rectangle sensors (107.45), as shown in Supplementary Fig. 30 and Supplementary Table 3. Furthermore, the minimum strain detection of flat and nanostructured serpentine sensors was 1% and 5%, respectively, which are much lower than those of our regular nanostructured sensors (0.015% and 0.005% at small (0%) and large (100%) strains, respectively). These observations lead us to conclude that the nanowire structure enhances both stretchability and sensitivity, and our methodology provides a complement to conventional geometric design of wearable electronics.

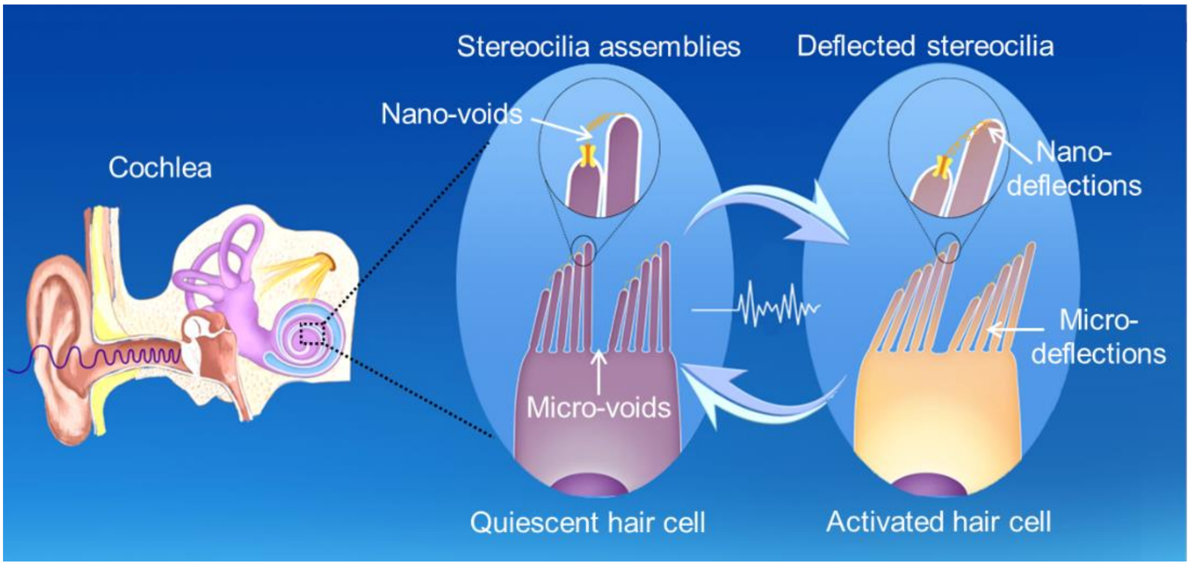

Supplementary Fig. 1 Process of acoustic signal detection by a cochlea.

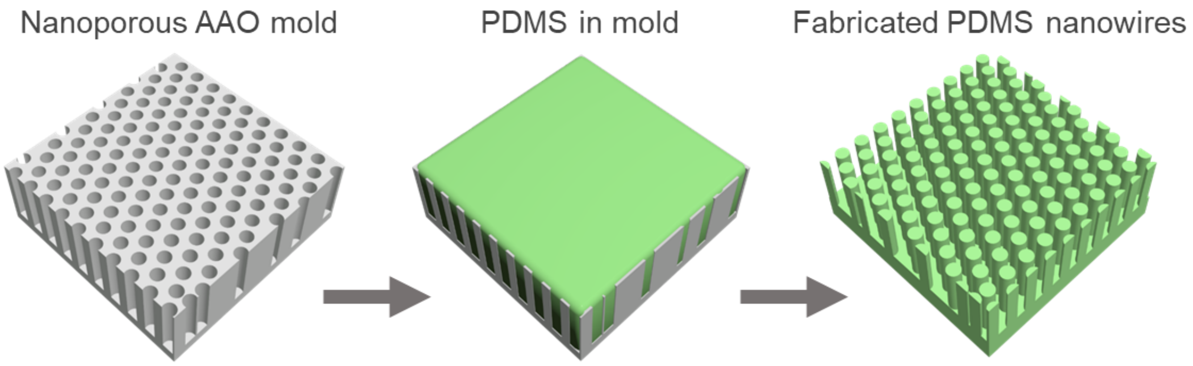

Supplementary Fig. 2 Fabrication of PDMS nanowires. PDMS precursors were first casted onto a porous AAO mold. After curing at high temperature, the mold was etched by adding acid to obtain PDMS nanowires.

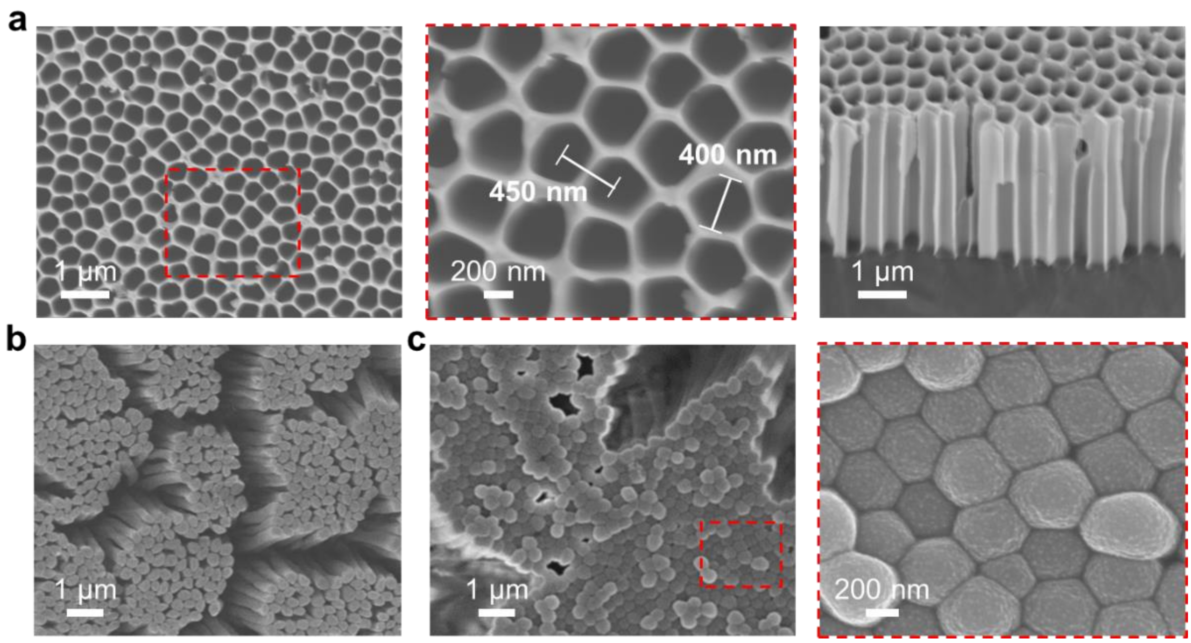

Supplementary Fig. 3 Morphology of AAO mold and formed nanowires. a SEM micrographs of the AAO mold. **b, c** SEM micrographs of the nanowires (**b**) before or (**c**) after assembling with ethanol. The AAO mold used to fabricate PDMS nanowire array has pores with a diameter of 400 nm and a center-to-center spacing of 450 nm. After removing the AAO mold in hydrogen chloride solutions and repeated washing in water, AAO-templated PDMS nanowire arrays with a diameter of 400 nm and a center-to-center spacing of 450 nm were formed. We notice here that the flexible PDMS nanowires were not closely bundled after removal of water. Next, we dispersed the nanowires in ethanol and subsequently evaporated the solvent to drive the assembly of PDMS nanowires. Finally, we measured the nanowire diameter and the center-to-center spacing to be the same value (400 nm) upon ethanol evaporation, suggesting formation of clusters of close-packed PDMS nanowires.

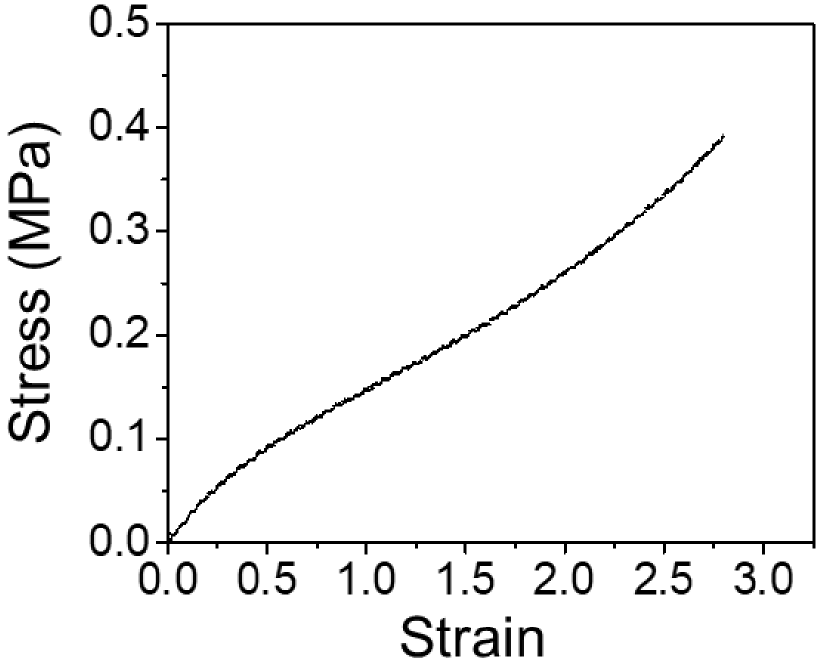

Supplementary Fig. 4 Stress-strain curve of PDMS substrate. According to the curve, we measured the Young's modulus of the PDMS substrate to be 0.24 MPa.

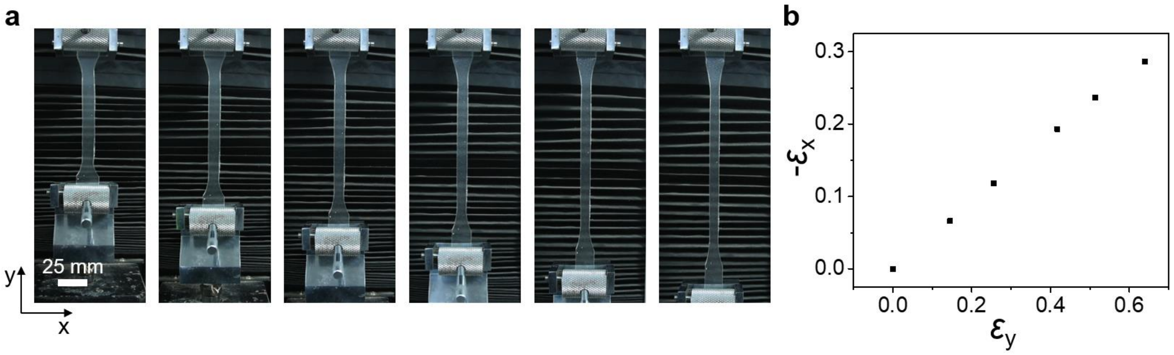

Supplementary Fig. 5 Poisson's ratio measurement. a Optical images of the stretched PDMS. **b** Axial strain (ε_x) and transverse strain (ε_y) calculated from the images. The Poisson's ratio was measured to be ~ 0.46 according to the requirements of determination of tensile properties in ISO 527-1:2012 [22, 23] (i.e., gauge length (75 mm), width in middle part (10 mm), thickness (4 mm), total length (170 mm), and test rate (1 mm/min)).

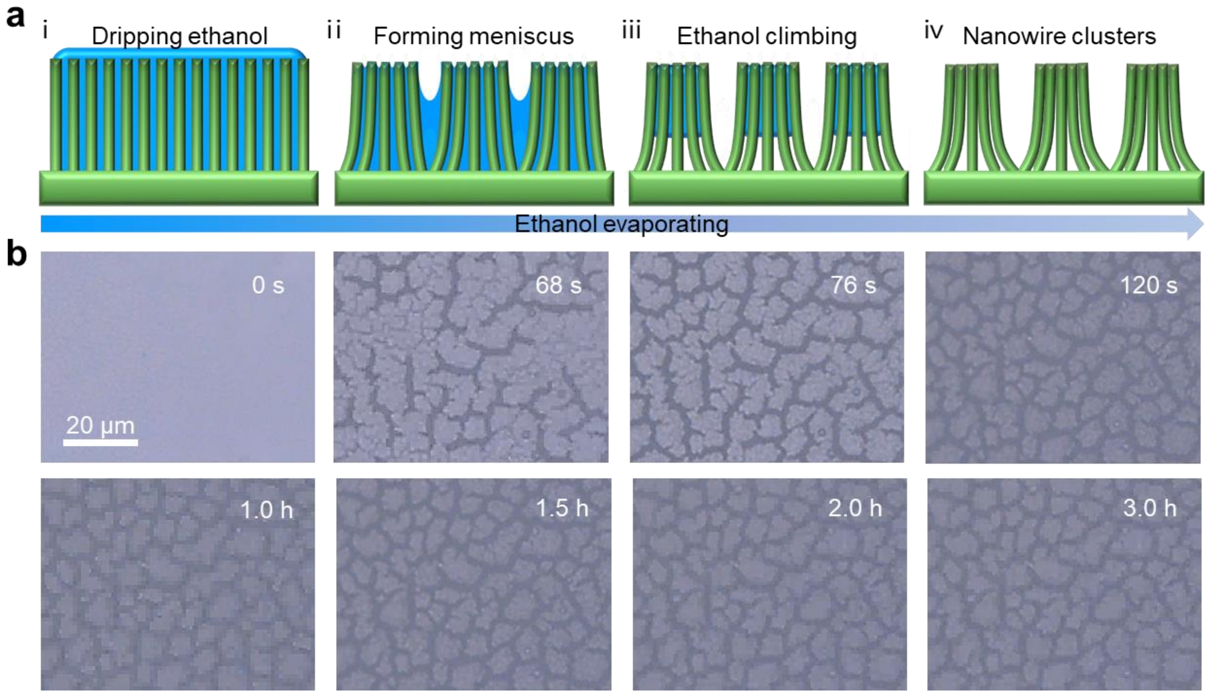

Supplementary Fig. 6 Temporal evolution of morphology of nanowire assemblies during ethanol evaporation. a Schematic illustration of assembling process of nanowires. **b** Morphology changes of assemblies during ethanol evaporation in 3 hours. Nanowires assembled into clusters within the first 120 s during the evaporation of ethanol, and no further change in the morphology of the formed nanowire assemblies was measured over the next 3 hours.

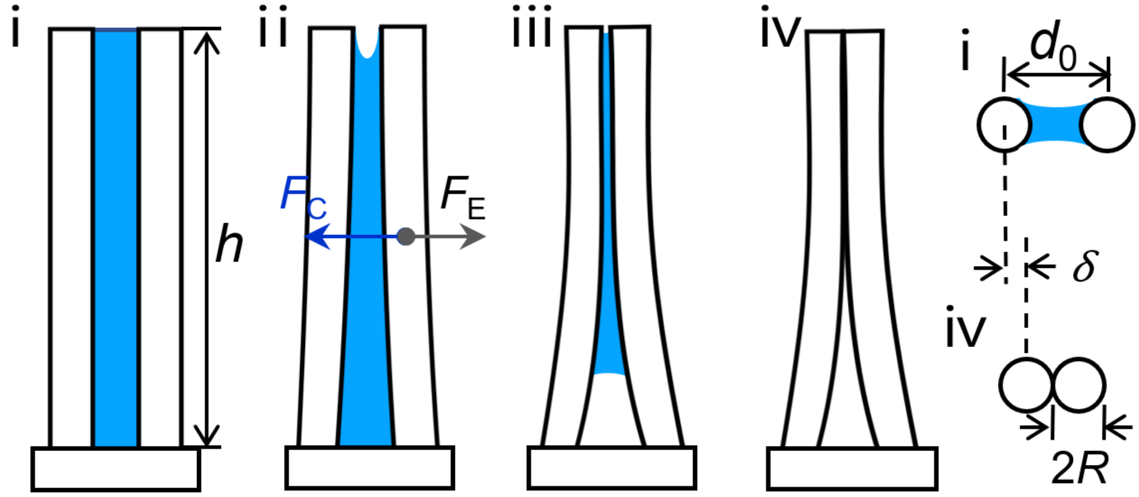

Supplementary Fig. 7 Force analysis during nanowire assembly process. See Supplementary Note 2 for detailed discussion.

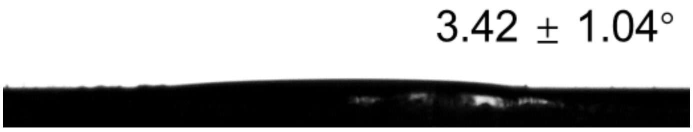

Supplementary Fig. 8 Optical image of ethanol on a flat PDMS surface. The intrinsic contact angle of ethanol on a flat PDMS surface is 3.42 ± 1.04°.

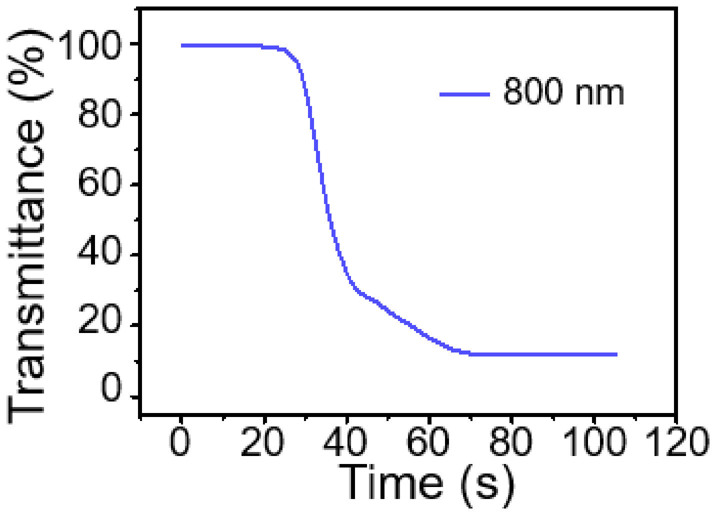

Supplementary Fig. 9 Optical transmittance of 4 μm-length nanowire-structured substrate as a function of aggregation state of nanowires. When dispersed in ethanol, the nanowires singly dispersed, resulting in high transparency. As ethanol evaporated, the nanowires self-assembled into clusters, giving rise to low optical transmittance due to light scattering.

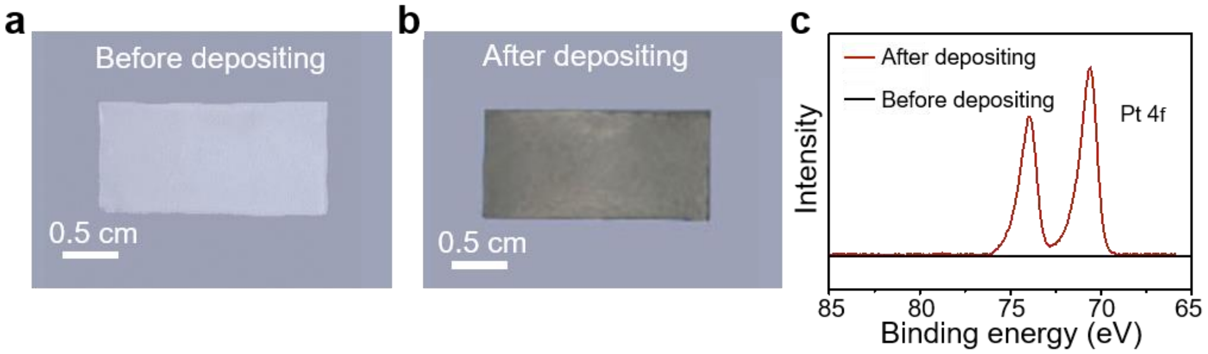

Supplementary Fig. 10 Deposition of nanometer-thick Pt film. a, b Optical appearance of nanowire-structured PDMS substrates before and after deposition of a 24 nm-thick Pt film. **c** Confirmation of the Pt deposition by XPS spectra, indicated by the characteristic Pt 4f peaks (i.e., 71.4 eV and 74.8 eV).

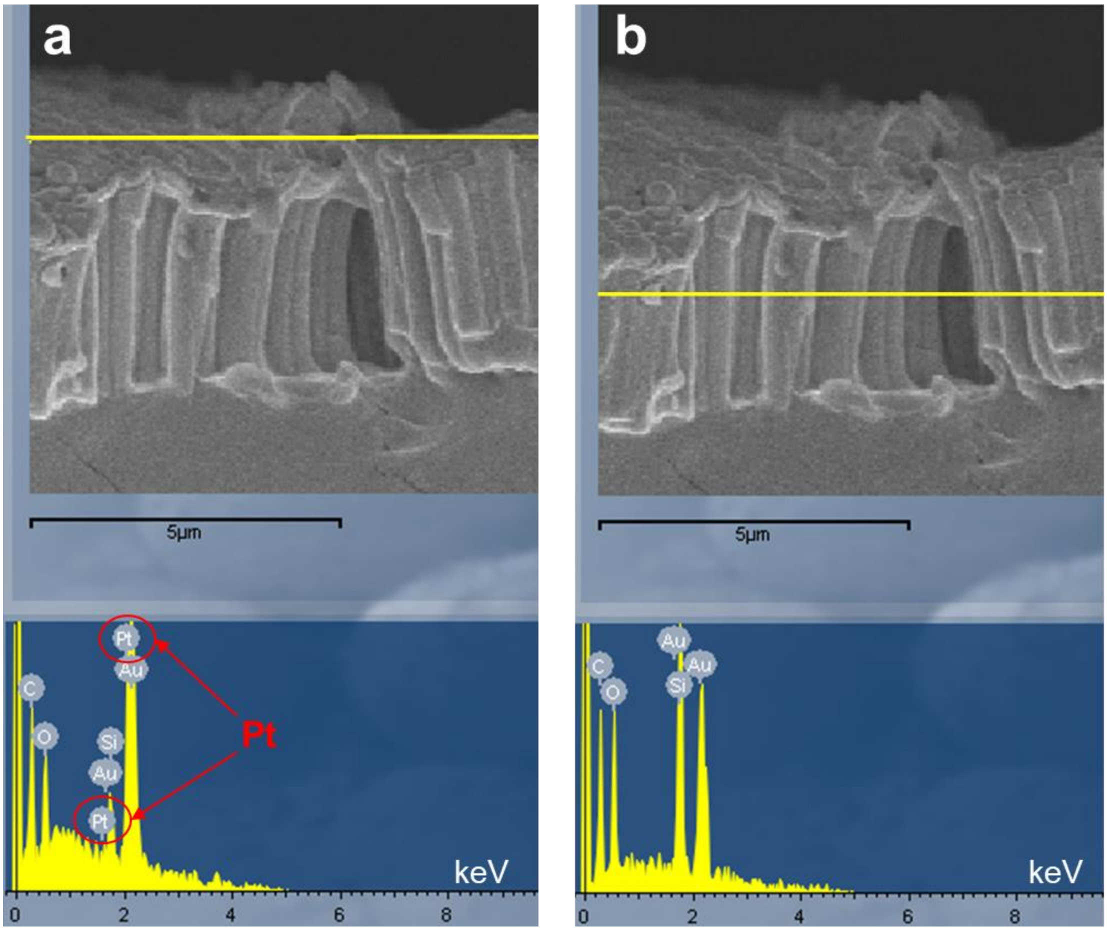

Supplementary Fig. 11 Element analysis of nanowire assemblies. a, b EDS scanning (**a**) at the top or (**b**) in the middle of the nanowire assemblies.

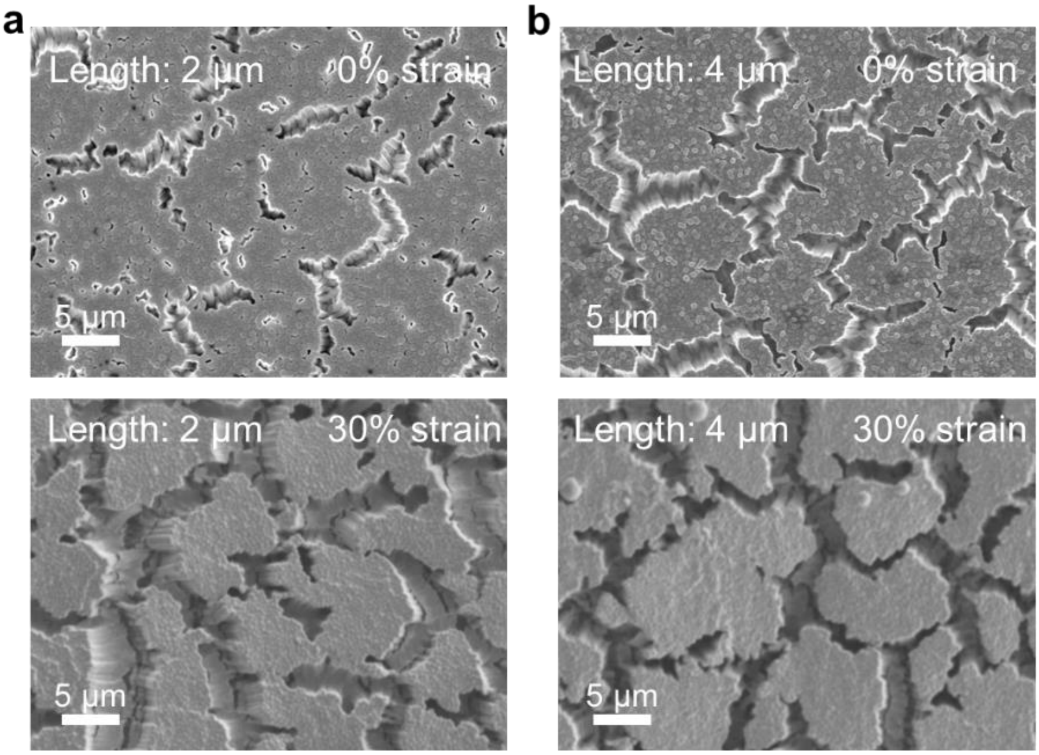

Supplementary Fig. 12 Influence of nanowire length on morphology of Pt-coated nanowire assemblies under 0% and 30% strain. a, b SEM images of 0% and 30% stretched Pt films coated on (**a**) 2 µm-length and (**b**) 4 µm-length nanowires, respectively. With the increase of the nanowire length, the width of the micro-voids increased (1.9 ± 0.3 µm and 2.4 ± 0.3 µm for 2 and 4 µm-length nanowire, respectively), and the numerical density of the micro-voids decreased. This is because longer nanowires are more bendable, resulting in bigger nanowire bundles with larger spacing. Upon an applied strain (e.g., 30%), the width of the cracks on the 2 µm-length nanowire-structured surfaces were 4.8 ± 0.6 µm, larger than that on 4 µm-length nanowire-structured surfaces (3.0 ± 0.4 µm) due to a weak crack retardation effect associated with short nanowires (the effect of nanowire-induced crack retardation has already been illustrated in Fig. 2 of the main text).

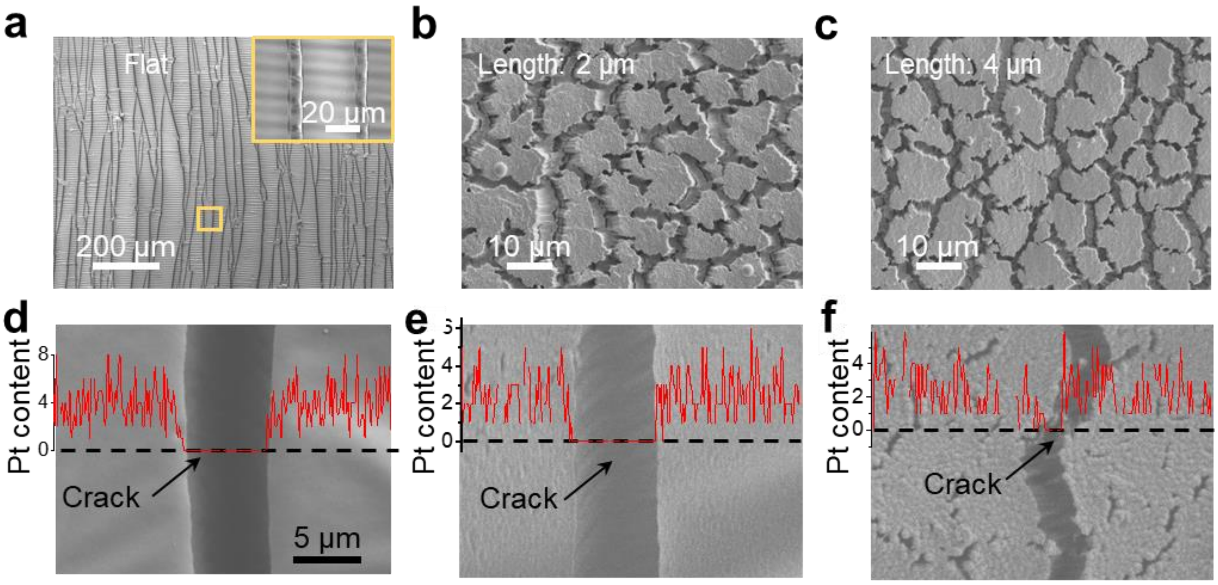

Supplementary Fig. 13 SEM micrographs and EDS curves of the cracks. SEM micrographs of the Pt films coated on a flat surface (**a, d**) or a surface consisting of clusters of 2 (**b, e**) or 4 μm-length (**c, f**) nanowires under 30% strain. The insets in **d-f** show Pt content obtained by EDS linear scanning along the dotted black line over the cracks.

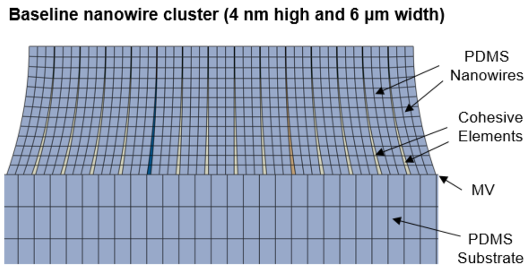

Specific properties of cohesive elements

Properties	C01	C02	C11	C12	C21	C22
Elastic	6e-7	1e-7	3e-7	5e-8	3.5e-7	6e-8
Maxs Damage	1.2e-7	1.6e-8	6e-8	8e-9	7e-8	1e-8
Damage Evolution	Displacement					
Displacement at failure	0.06	0.02	0.06	0.02	0.06	0.02
Viscosity	0.0001					
Tolerance	0.05					

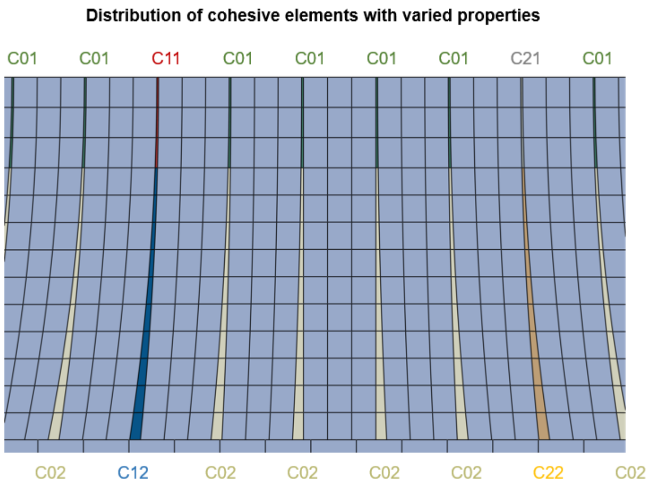

Supplementary Fig. 14 Distribution and properties of cohesive elements in a nanowire cluster.

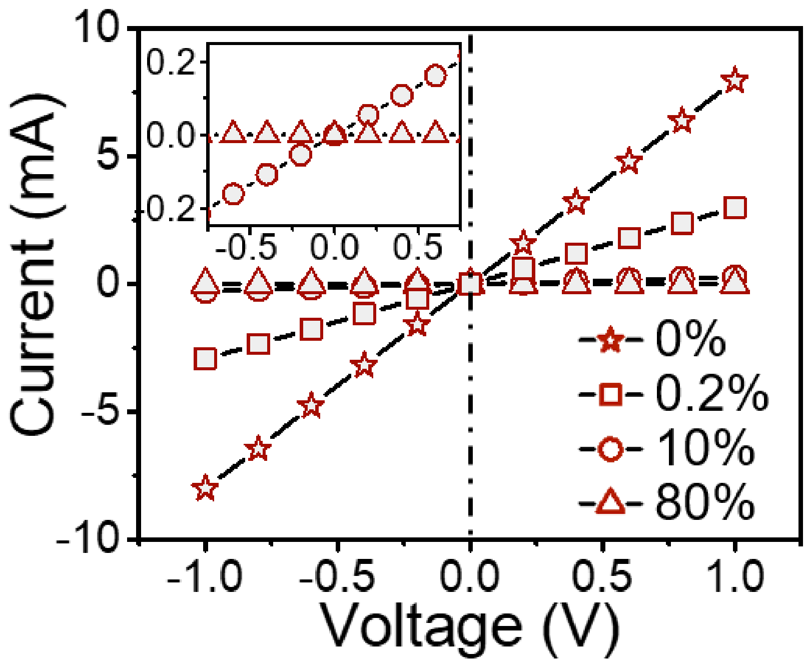

Supplementary Fig. 15 I-V curve of Pt films under strains. Resistance of the Pt film under different strains can be calculated from the I-V curves.

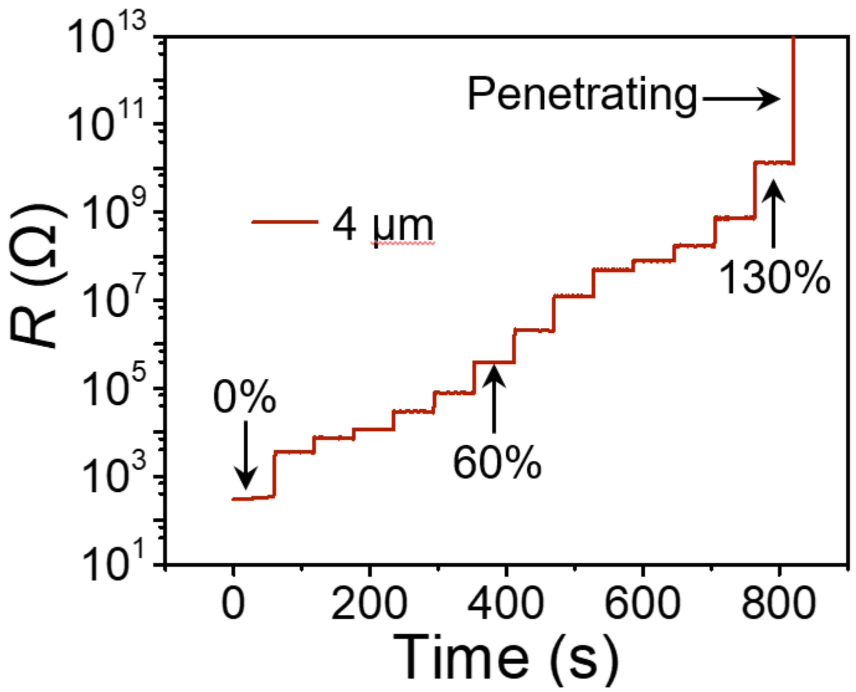

Supplementary Fig. 16 Resistance of 4 μm-length nanowires-structured Pt films as a function of strain. We note here that above 130% strain, penetrating cracks dramatically increase the resistance and cause the device to fail. Each step represents an increase of strain by 10%.

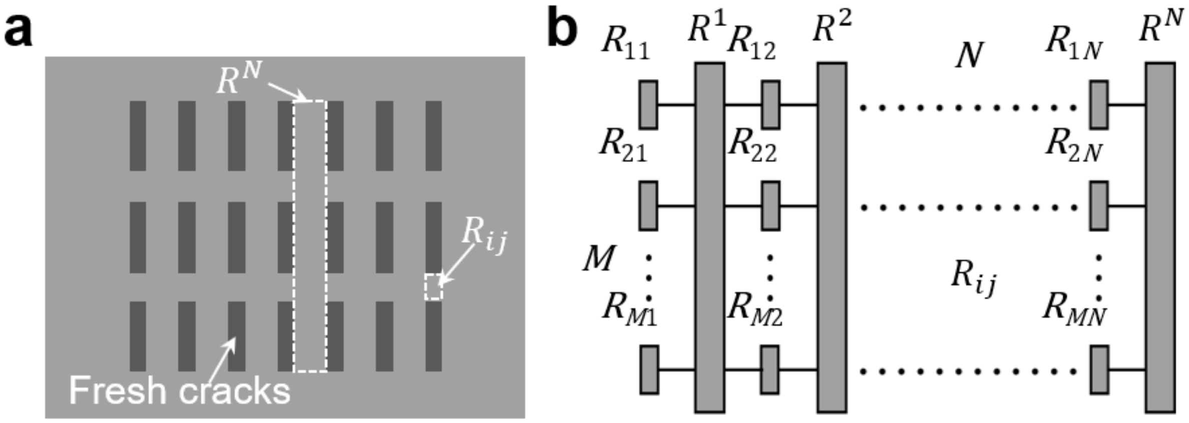

Supplementary Fig. 17 Model for calculation of resistance of a cracked Pt film. a In our model, strain-induced cracks were assumed to be evenly distributed over the Pt film. **b** Equivalent circuits for the calculation of the resistance of the Pt film. See Supplementary Note 4 for detailed discussion.

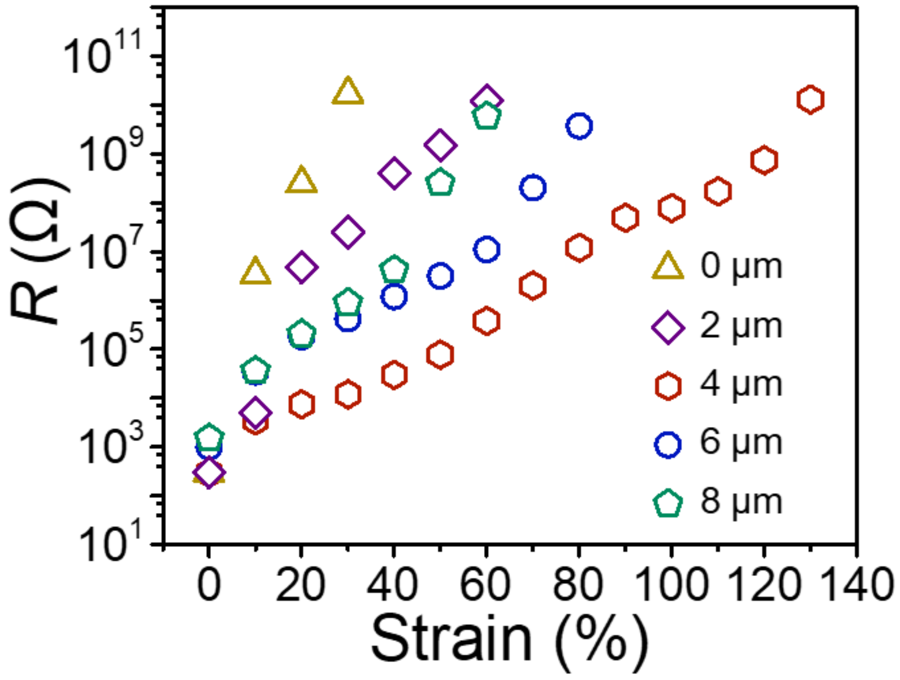

Supplementary Fig. 18 Electric resistance of Pt films as a function of strains. The sensor with 4 μm-length nanowires exhibited the largest tolerable strain.

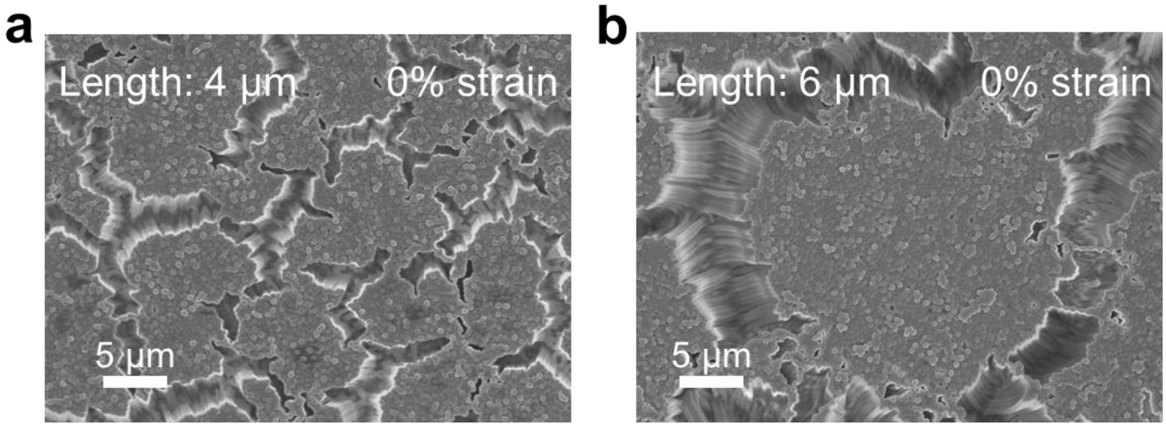

Supplementary Fig. 19 SEM micrographs of micro-voids on nanowire assemblies. a SEM images of micro-voids formed on 4 μm-length nanowire assemblies. **b** SEM images of micro-voids formed on 6 μm-length nanowire assemblies. Inspection of Supplementary Fig. 19 reveals that longer nanowires caused larger micro-voids.

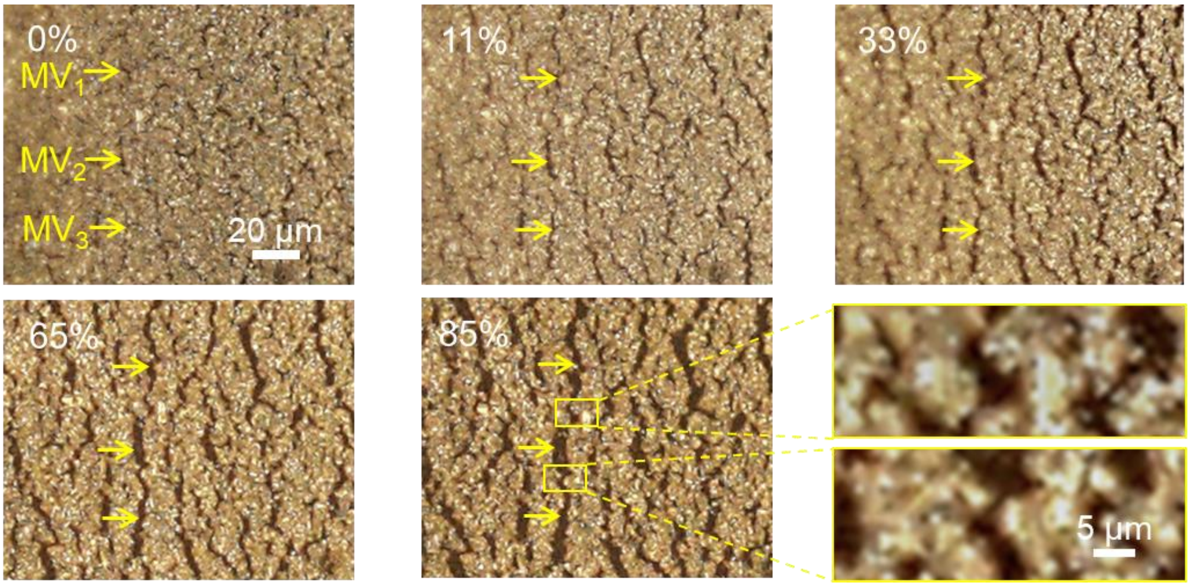

Supplementary Fig. 20 In-situ imaged cracking process of Pt films coated on surface with 6 µm–length nanowire assemblies at different strains. At the surfaces with 6 µm-length nanowire assemblies, the first micro-void-initiated cracking stage was deferred to ~65%, and the micro-voids (e.g., MV_1, MV_2 and MV_3) induced much longer cracks than that of 4 µm-length nanowire-structured surfaces, resulting in crack penetration at the strain of ~ 80%.

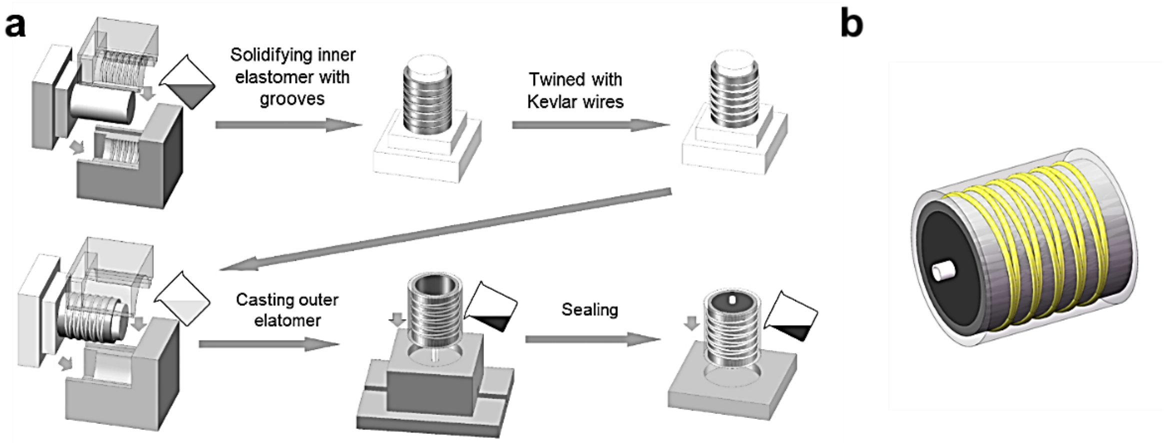

Supplementary Fig. 21 Fabrication of 1D actuator. Reproduced with permission from REF. 24, the American Association for the Advancement of Science. **a** Scheme of fabrication procedure of 1D actuator. First, inner elastomeric core with grooves was fabricated by solidifying a silicone elastomer in a home-made mold. Second, Kevlar wires were twined into the grooves. Third, the outer elastomer shell was cured to sandwich the Kevlar wires into the elastomer. Finally, side walls were fabricated to seal the air chamber. **b** Schematic illustration of the fabricated 1D actuator. Kevlar wires were used to convert variation of air pressure into changes in its length along the long axis of the actuator.

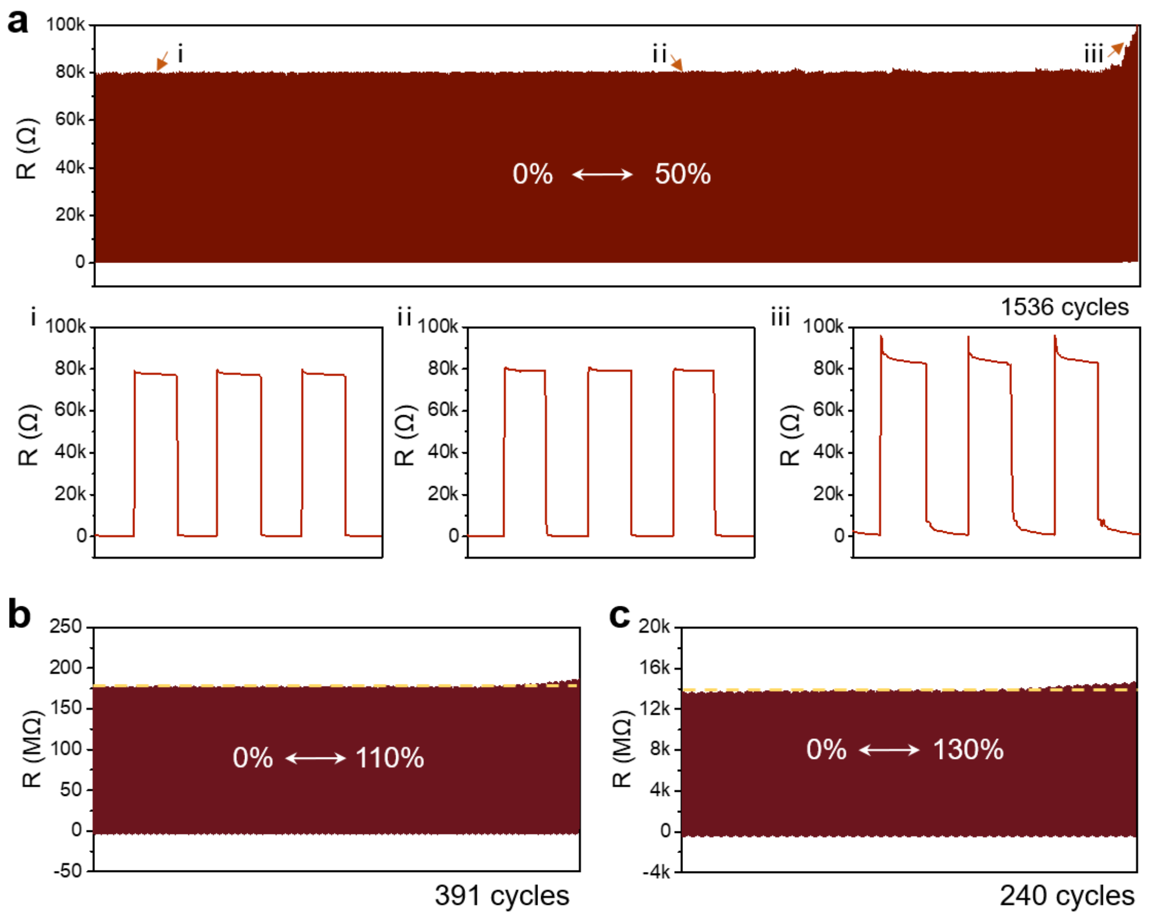

Supplementary Fig. 22 Durability of sensor. a, b, c Cyclic test at strain of (**a**) 50%, (**b**) 110%, (**c**) 130%, respectively. Insets in **a**:i, ii, iii were three close-ups of typical cycles indicated by the arrows.

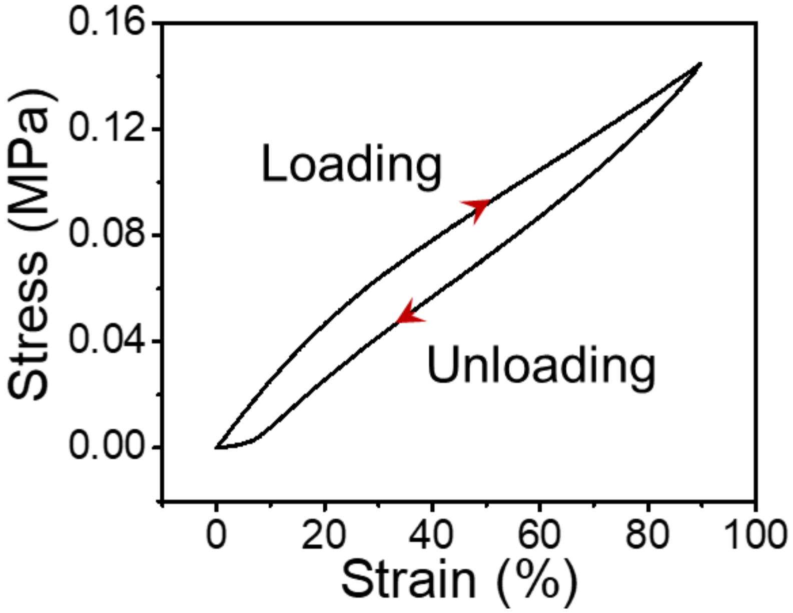

Supplementary Fig. 23 Hysteresis curve of PEIE-modified PDMS.

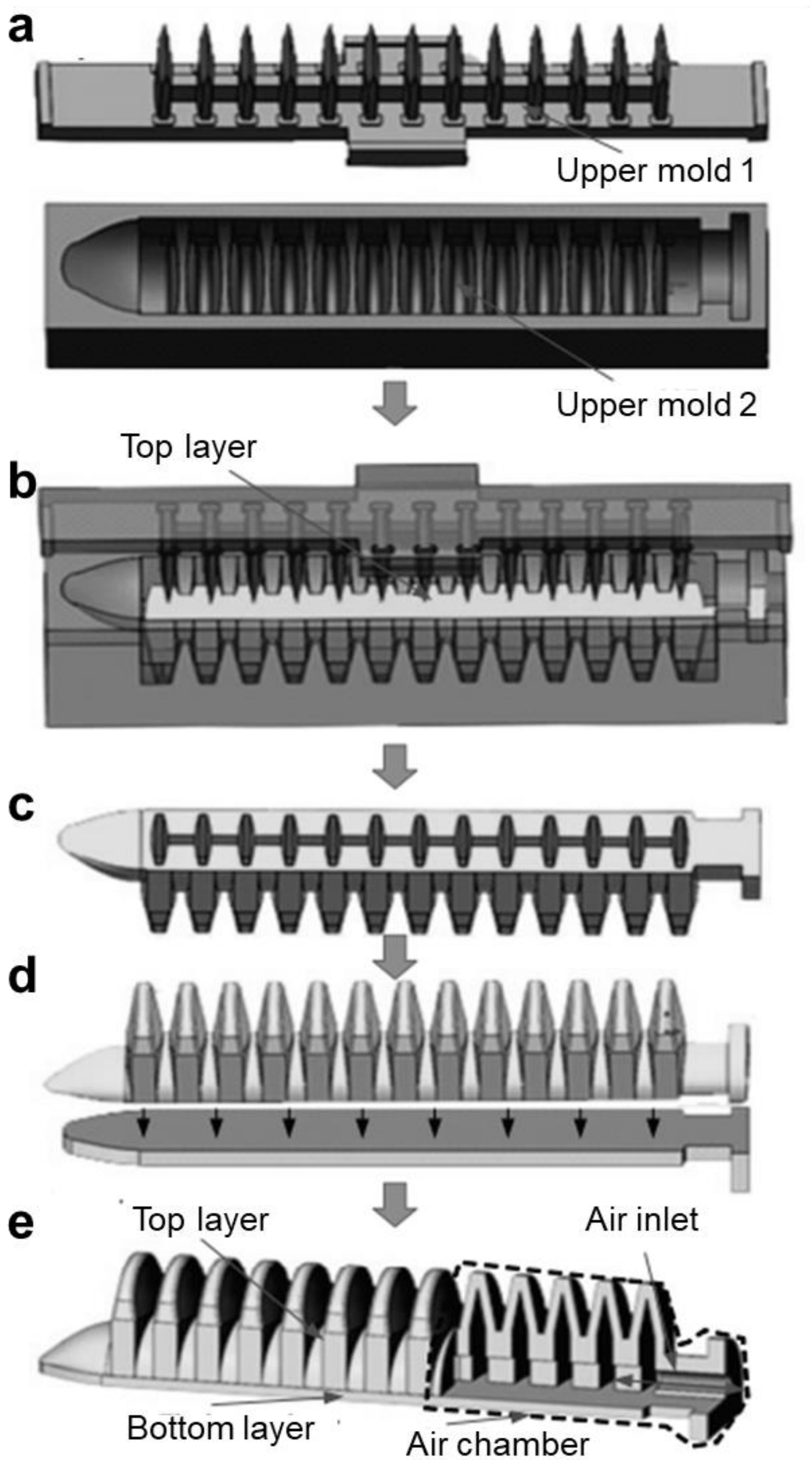

Supplementary Fig. 24 Fabrication of a soft gripper. Reproduced with permission from REF. 25, Macmillan Publishers Limited. **a, b, c** The silicone elastomer was molded into the top layers of a soft gripper. **d, e** The stretchable top layer and non-stretchable bottom layer were glued together to form an air chamber inside the gripper.

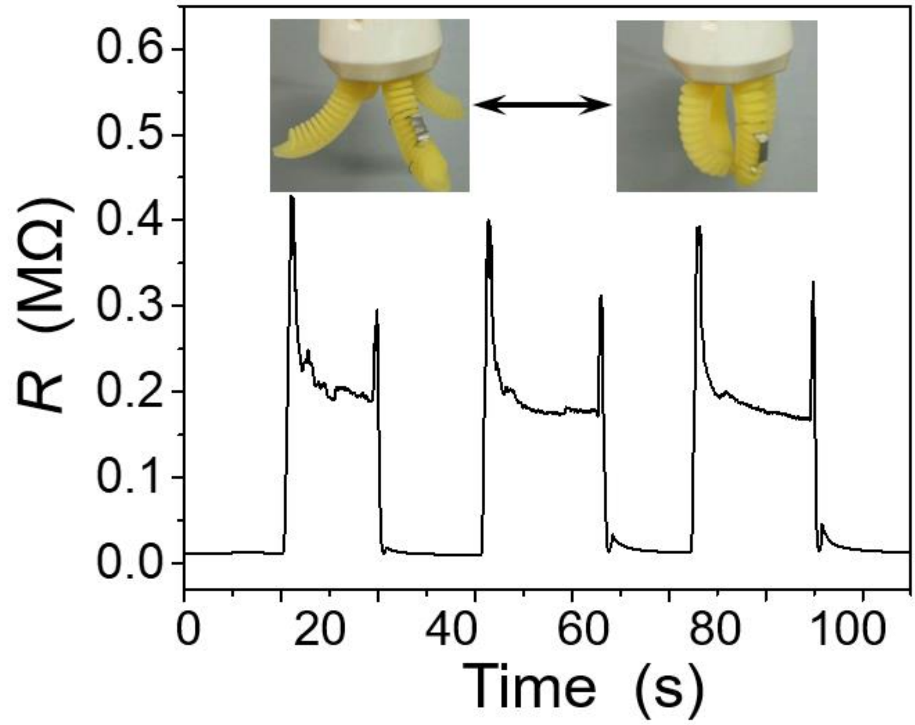

Supplementary Fig. 25 Response of nanowire-structured Pt film-based electronic sensor to different types of motions of soft gripper. The motions contain repeating grabbing, moving and releasing of an object.

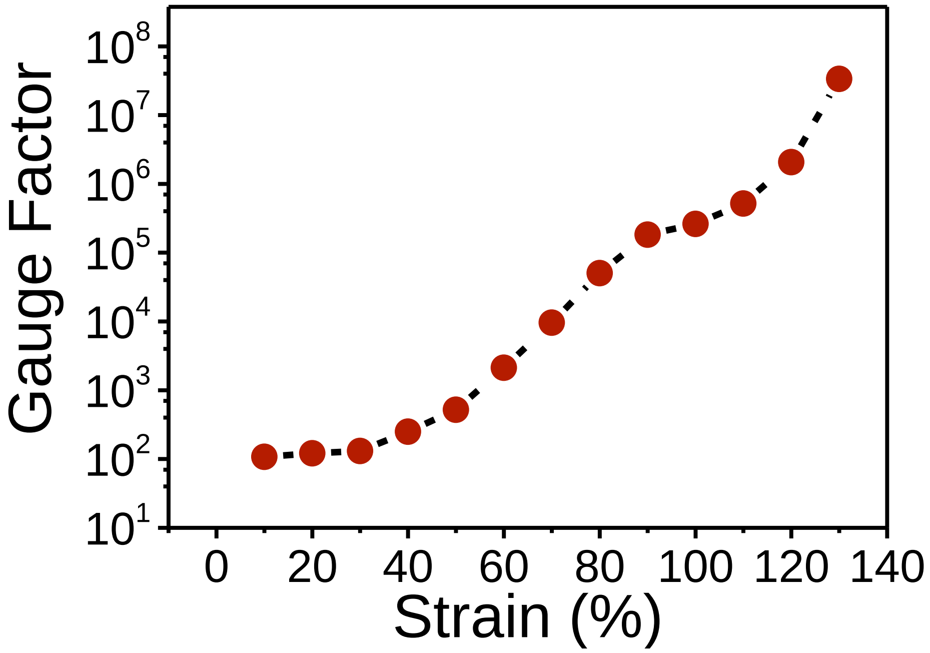

Supplementary Fig. 26 Gauge factor of Pt film-based electronic device as a function of strain. Gauge factor is calculated as $\Delta R/(\varepsilon R_0)$, in which ε represents the applied strain.

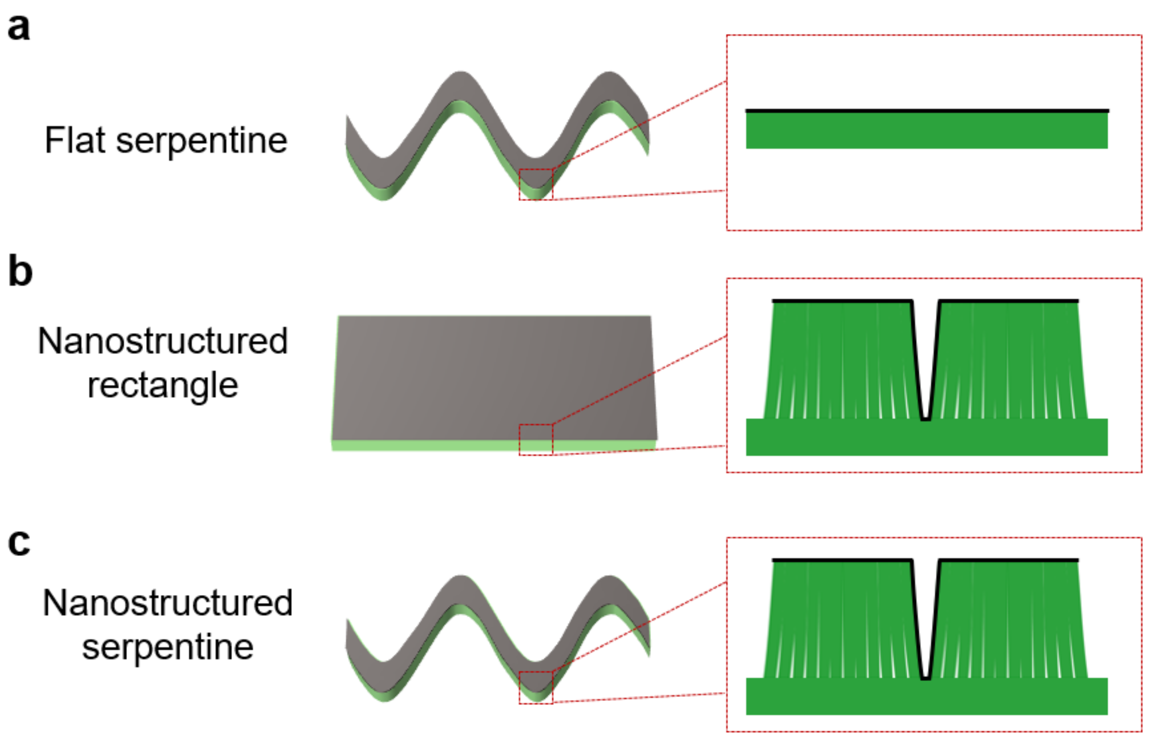

Supplementary Fig. 27 Sensors with different macroscopic geometrical shapes and microscopic surface structures. a Serpentine sensor with Pt film deposited on flat surface (flat serpentine sensor). **b** Serpentine sensor with Pt film deposited on 4 μm-length nanowire assemblies (nanostructured rectangle sensor). **c** Rectangle sensor with Pt film deposited on 4 μm-length nanowire assemblies (nanostructured serpentine sensor).

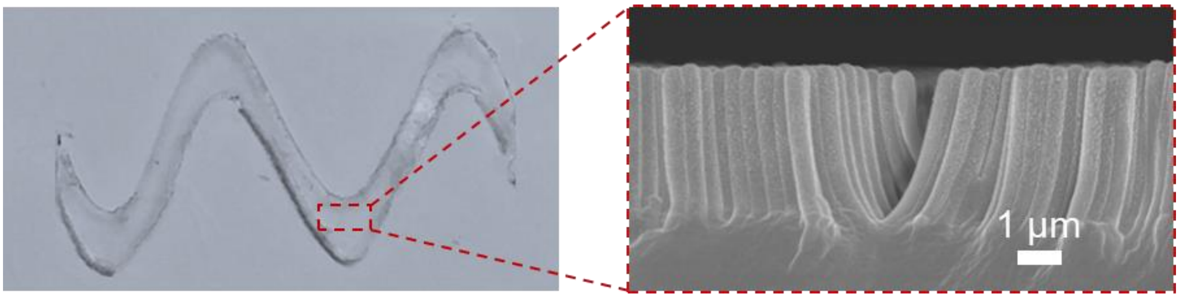

Supplementary Fig. 28 Optical microscopy image and SEM micrograph of nanostructured serpentine sensor.

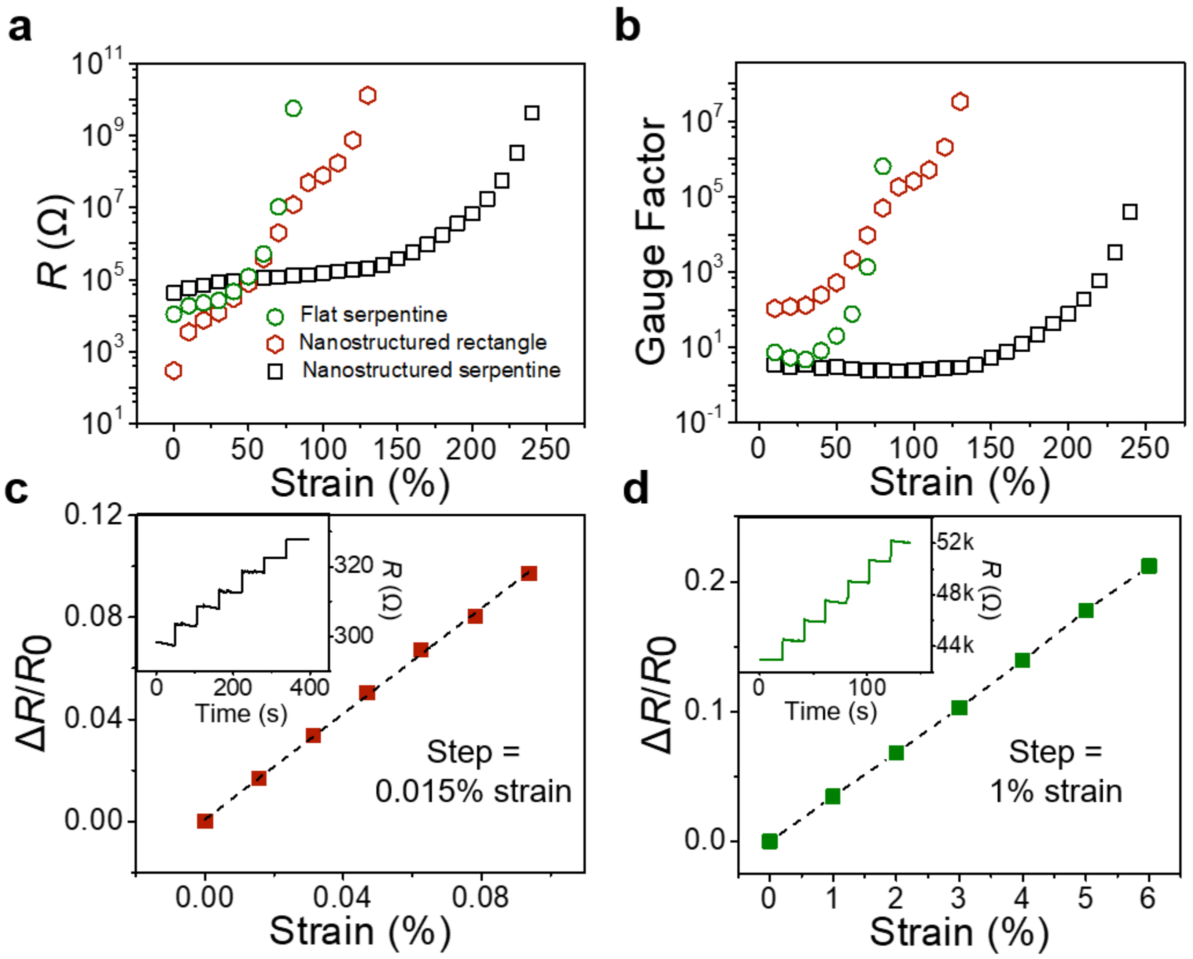

Supplementary Fig. 29 Influence of geometrical shape on electric resistance of metal film-based sensors. a Electrical resistance and gauge factor of the sensors as a function of strain, indicating that our nanostructured rectangle sensor has a higher gauge factor value than serpentine sensors. **b, c** $\Delta R/R_0$ of the nanostructured rectangle sensor and the flat serpentine sensor versus strain. Insets show the corresponding resistance as a function of time.

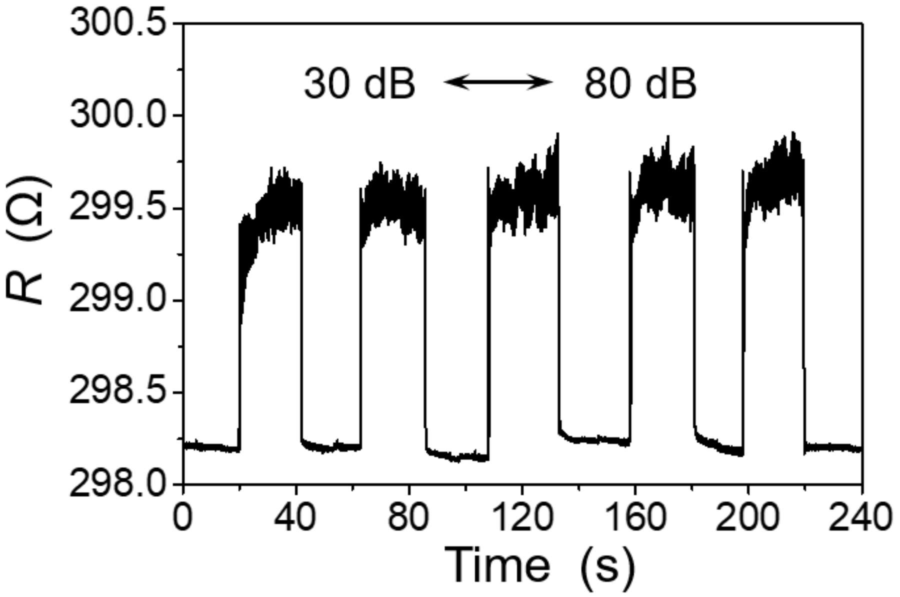

Supplementary Fig. 30 Resistance of metal film sensors as a function of repeating sounds between 30 dB and 80 dB. The resistance change corresponds to the Supplementary Movie 6, showing the good reversibility of our sensors in the detection of sounds.

Supplementary Table 1. Deformation near micro-voids and global strain during nano-void-guided-cracking process.

Deformation measured from 600 nm above the micro-void (Unit: nm)						
		Cluster width (μm)				
		6	7	8	9	10
Cluster height (μm)	2	531	473	431	386	399
	4	547	576	615	612	561
	6	645	828	771	710	726
	8	947	925	857	803	752
	10	981	909	830	764	823
Global strain of the film with NV-governed process (Unit: %)						
		Cluster width (μm)				
		6	7	8	9	10
Cluster height (μm)	2	48.8	43.4	33.3	33.6	32.9
	4	67.2	64.2	62.8	54.4	43.6
	6	107.6	93	93.8	86.5	81.4
	8	137.6	125	118.4	113.3	98.6
	10	147.6	149.5	151.2	146.7	140.2

Supplementary Table 2. Sensitivity of metal material-based stretchable sensors and other piezoresistive stretchable sensors.

Sensor	Maximum strain range	Gauge factor (strain)	Minimum detection (strain)
Au nanobelt-based sensor[26]	130%	~10	2.5%
Au mesh-based sensor[27]	130%	<5	—
Ag nanoparticle-based sensor[28]	140%	10	2%
Carbon nanotube/conducting polymer-based sensor[29]	100%	62	1.6%
Carbon nanotubes and graphite films-based sensor[30]	620%	43	8.3%
Carbon nanotube/ polyurethane-based sensor[31]	400%	69	15.5%
Carbon nanotube/Ecoflex[32]	400%	70.2	—
Pt film-based sensor[33]	2%	16,000	~0.5%
Ag plated nylon-based sensor[34]	75%	~10	1%
Ag nanowire-based sensor[35]	60%	150,000	5%
Nanowire-structured Pt film-based sensor (this work)	130%	107.45	0.015% (0%)<br>0.005% (100%)

Supplementary Table 3. Performance of metal film-based ultrastretchable sensors in different geometrical shapes.

Geometry	Conducting material	Maximum strain range	Sensitivity (Gauge Factor)	Minimum detection
**Serpentine**[36]	Cu film	81%	—	—
**Serpentine**[37]	Au&Ag film	120%	<1	—
**Sinusoidal structures**[26]	Au Nanobelt	130%	≈10	2.5%
**Fracture network**[27]	Au mesh	130%	<5	—
**Mesh network**[28]	Ag nanoparticle	140%	10	2%
**Mesh network**[38]	Ag Nanowire	460%	≈1	—
**Binary network**[39]	Ag Nanowire	100%	≈1	—
**Our flat serpentine sensor**	Pt film	80%	7.34	1%
**Our nanostructured serpentine sensor**	Pt film	240%	3.53	5%
**Our nanostructured rectangle sensor**	**Pt film**	**130%**	**107.45**	**0.015% (0%)** **0.005% (100%)**

Supplementary References

1. Hudspeth, A. J. Integrating the active process of hair cells with cochlear function. *Nat. Rev. Neurosci.* **15**, 600-614 (2014).
2. Lee, J.-H. et al. Magnetic nanoparticles for ultrafast mechanical control of inner ear hair cells. *ACS Nano* **8**, 6590-6598 (2014).
3. Slepecky, N. Overview of mechanical damage to the inner ear: Noise as a tool to probe cochlear function. *Hearing Res.* **22**, 307-321 (1986).
4. Poche, L. B., Stockwell, C. W., Ades, H. W. Cochlear hair-cell damage in guinea pigs after exposure to impulse noise. *J. Acoust. Soc. Am.* **46**, 947-951 (1969).
5. Oishi, N., Schacht, J. Emerging treatments for noise-induced hearing loss. *Expert Opin. Emerging Drugs* **16**, 235-245 (2011).
6. Chandra, D., Yang, S. Capillary-force-induced clustering of micropillar arrays: Is it caused by isolated capillary bridges or by the lateral capillary meniscus interaction force? *Langmuir* **25**, 10430-10434 (2009).
7. Duprat, C., Protiere, S., Beebe, A. Y., Stone, H. A. Wetting of flexible fibre arrays. *Nature* **482**, 510-513 (2012).
8. Rieser, J. M. et al. Tunable capillary-induced attraction between vertical cylinders. *Langmuir* **31**, 2421-2429 (2015).
9. Sidorenko, A. et al. Reversible switching of hydrogel-actuated nanostructures into complex micropatterns. *Science* **315**, 487-490 (2007).
10. Alfadhel, A., Kosel, J. Magnetic nanocomposite cilia tactile sensor. *Adv. Mater.* **27**, 7888-7892 (2015).
11. Yao, X. et al. Adaptive fluid-infused porous films with tunable transparency and wettability. *Nat. Mater.* **12**, 529-534 (2013).
12. She, X., Shen, Y., Wang, J., Jin, C. Pd films on soft substrates: A visual, high-contrast and low-cost optical hydrogen sensor. *Light Sci. Appl.* **8**, 4 (2019).
13. Chang, J.-B. et al. Design rules for self-assembled block copolymer patterns using tiled templates. *Nat. Commun.* **5**, 3305 (2014).
14. Tavakkoli, A. K. G. et al. Multilayer block copolymer meshes by orthogonal self-assembly. *Nat. Commun.* **7**, 10518 (2016).
15. Svetlizky, I. et al. Properties of the shear stress peak radiated ahead of rapidly accelerating rupture fronts that mediate frictional slip. *Proc. Natl. Acad. Sci. U. S. A.* **113**, 542-547 (2016).
16. Wie, D. S. et al. Wafer-recyclable, environment-friendly transfer printing for large-scale thin-film nanoelectronics. *Proc. Natl. Acad. Sci. U. S. A.* **115**, E7236-E7244 (2018).
17. Hernandez, E., Alfano, M., Pulungan, D., Lubineau, G. Toughness amplification in copper/epoxy joints through pulsed laser micro-machined interface heterogeneities. *Sci. Rep.* **7**, 16344 (2017).
18. Lacour, S. P. et al. Mechanisms of reversible stretchability of thin metal films on elastomeric substrates. *Appl. Phys. Lett.* **88**, 3 (2006).

19. Graz, I. M., Cotton, D. P. J., Lacour, S. P. Extended cyclic uniaxial loading of stretchable gold thin-films on elastomeric substrates. *Appl. Phys. Lett.* **94**, 3 (2009).
20. Lee, J. et al. A stretchable strain sensor based on a metal nanoparticle thin film for human motion detection. *Nanoscale* **6**, 11932-11939 (2014).
21. Kang, D. et al. Ultrasensitive mechanical crack-based sensor inspired by the spider sensory system. *Nature* **516**, 222-226 (2014).
22. ISO 527-1:2012 Plastics -- Determination of tensile properties -- Part 1: General principles.
23. ISO 527-2:2012 Plastics -- Determination of tensile properties -- Part 2: Test conditions for moulding and extrusion plastics.
24. Wang, Y. et al. A biorobotic adhesive disc for underwater hitchhiking inspired by the remora suckerfish. *Sci. Robot,* **2**, 9 (2017).
25. Hao, Y. F. et al. A soft bionic gripper with variable effective length. *J. Bionic. Eng.* **15**, 220-235 (2018).
26. Qi, D. et al. Highly stretchable gold nanobelts with sinusoidal structures for recording electrocorticograms. *Adv. Mater.* **27**, 3145-3151 (2015).
27. Ho, M. D. et al. Fractal gold nanoframework for highly stretchable transparent strain-insensitive conductors. *Nano Lett.* **18**, 3593-3599 (2018).
28. Park, M. et al. Highly stretchable electric circuits from a composite material of silver nanoparticles and elastomeric fibres. *Nat. Nanotechnol.* **7**, 803-809 (2012).
29. Roh, E. et al. Stretchable, transparent, ultrasensitive, and patchable strain sensor for human-machine interfaces comprising a nanohybrid of carbon nanotubes and conductive elastomers. *ACS Nano* **9**, 6252-6261 (2015).
30. Tadakaluru, S., Thongsuwan, W., Singjai, P. Stretchable and flexible high-strain sensors made using carbon nanotubes and graphite films on natural rubber. *Sensors* **14**, 868-876 (2014).
31. Slobodian, P. et al. A flexible multifunctional sensor based on carbon nanotube/polyurethane composite. *IEEE Sens. J.* **13**, 4045-4048 (2013).
32. Jiang, Y. T. et al. Stretchable CNTs-ecoflex composite as variable-transmittance skin for ultrasensitive strain sensing. *Adv. Mater. Technol.* **3**, 7 (2018).
33. Park, B. et al. Dramatically enhanced mechanosensitivity and signal-to-noise ratio of nanoscale crack-based sensors: Effect of crack depth. *Adv. Mater.* **28**, 8130-8137 (2016).
34. Tolvanen, J., Hannu, J., Jantunen, H. Stretchable and washable strain sensor based on cracking structure for human motion monitoring. *Sci. Rep.* **8**, 10 (2018).
35. Liao, X. et al. Ultrasensitive and stretchable resistive strain sensors designed for wearable electronics. *Mater. Horiz.* **4**, 502-510 (2017).
36. Zhang, Y. et al. Buckling in serpentine microstructures and applications in elastomer-supported ultra-stretchable electronics with high areal coverage. *Soft Matter* **9**, 8062-8070 (2013).
37. Jang, S. et al. A high aspect ratio serpentine structure for use as a strain-insensitive, stretchable transparent conductor. *Small* **14**, 9 (2018).

38. Lee, P. et al. Highly stretchable and highly conductive metal electrode by very long metal nanowire percolation network. *Adv. Mater.* **24**, 3326-3332 (2012).
39. Ge, J. et al. Stretchable conductors based on silver nanowires: Improved performance through a binary network design. *Angew. Chem. Int. Ed.* **52**, 1654-1659 (2013).
