# Supplementary material for: Micro-/nano-voids guided two-stage film cracking on bioinspired assemblies for high-performance electronics
Source: Nat Commun. 2019 Aug 27;10:3862. doi: 10.1038/s41467-019-11803-8 (PMC6711965; doi:10.1038/s41467-019-11803-8)
Supplement: Supplementary file 2 — Description of Additional Supplementary Files [file 41467_2019_11803_MOESM2_ESM.docx]

Description of Additional Supplementary Files

**Supplementary Movie 1.** Real-time formation of bioinspired hierarchical assembly of nanowires driven by ethanol evaporation. The length of the nanowire is 4 µm.

**Supplementary Movie 2.** Finite element simulation of cracking process of nanowire-structured surfaces. The length of the nanowire is 4 µm.

**Supplementary Movie 3.** Reversibility of nanowire-structured Pt film-based motion detector. The length of the nanowire is 4 µm.

**Supplementary Movie 4.** Nanowire-structured Pt film for detection of soft gripper’s locomotion. The length of the nanowire is 4 µm.

**Supplementary Movie 5.** Influence of sound intensity on resistance of nanowire-structured Pt film-based sound detector. The sound intensities tested in the movie are 63 dB, 69 dB and 79 dB. The length of the nanowire is 4 µm.

**Supplementary Movie 6.** Reversibility of nanowire-structured Pt film-based sound detector. The intensity of the acoustic signal in the movie changed between 30 dB and 80 dB periodically. The length of the nanowire is 4 µm.
