# Supplementary figures and images for: Micro-/nano-voids guided two-stage film cracking on bioinspired assemblies for high-performance electronics
Source: Nat Commun. 2019 Aug 27;10:3862. doi: 10.1038/s41467-019-11803-8 (PMC6711965; doi:10.1038/s41467-019-11803-8)

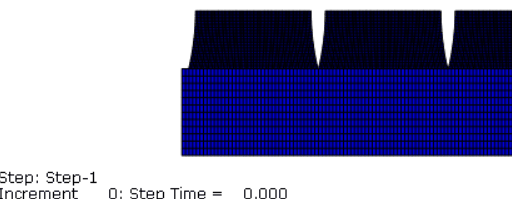

Supplement: Supplementary file 4 — Supplementary Movie 2 [file 41467_2019_11803_MOESM4_ESM.gif]
